# Contribution of Oxidative Stress and Impaired Biogenesis of Pancreatic β-Cells to Type 2 Diabetes

**DOI:** 10.1089/ars.2018.7656

**Published:** 2019-08-22

**Authors:** Petr Ježek, Martin Jabůrek, Lydie Plecitá-Hlavatá

**Affiliations:** Department of Mitochondrial Physiology, Institute of Physiology of the Czech Academy of Sciences, Prague, Czech Republic.

**Keywords:** pancreatic β-cells, type 2 diabetes, oxidative stress, impaired redox signaling, β-cell identity self-checking, dedifferentiation

## Abstract

***Significance:*** Type 2 diabetes development involves multiple changes in β-cells, related to the oxidative stress and impaired redox signaling, beginning frequently by sustained overfeeding due to the resulting lipotoxicity and glucotoxicity. Uncovering relationships among the dysregulated metabolism, impaired β-cell “well-being,” biogenesis, or cross talk with peripheral insulin resistance is required for elucidation of type 2 diabetes etiology.

***Recent Advances:*** It has been recognized that the oxidative stress, lipotoxicity, and glucotoxicity cannot be separated from numerous other cell pathology events, such as the attempted compensation of β-cell for the increased insulin demand and dynamics of β-cell biogenesis and its “reversal” at dedifferentiation, that is, from the concomitantly decreasing islet β-cell mass (also due to transdifferentiation) and low-grade islet or systemic inflammation.

***Critical Issues:*** At prediabetes, the compensation responses of β-cells, attempting to delay the pathology progression—when exaggerated—set a new state, in which a self-checking redox signaling related to the expression of *Ins* gene expression is impaired. The resulting altered redox signaling, diminished insulin secretion responses to various secretagogues including glucose, may lead to excretion of cytokines or chemokines by β-cells or excretion of endosomes. They could substantiate putative stress signals to the periphery. Subsequent changes and lasting glucolipotoxicity promote islet inflammatory responses and further pathology spiral.

***Future Directions:*** Should bring an understanding of the β-cell self-checking and related redox signaling, including the putative stress signal to periphery. Strategies to cure or prevent type 2 diabetes could be based on the substitution of the “wrong” signal by the “correct” self-checking signal.

## Introduction

### Type 2 diabetes etiology

#### Consensus on type 2 diabetes etiology

Traditionally, it is emphasized that insulin stimulates the disposal of glucose into the skeletal muscle and white adipose tissue (WAT), reduces glycogenolysis and gluconeogenesis in the liver, and suppresses lipolysis in WAT (30). Insulin resistance (IR) is consequently viewed as the inability of the above events or their reduction, such as insufficient glucose uptake into skeletal muscle, enhanced glucose release from the liver, and enhanced supply of free fatty acids (FAs) to the blood from WAT. Typically, during the glucose tolerance test, IR is manifested by the delayed response on glucose. For healthy individuals, after a glucose bolus, one can recognize the typical high and sharp first phase of insulin release, followed by the second phase with about a half amplitude and prolonged decay (up to 1 hour).

At the progressed type 2 diabetes, the first phase is often missing, whereas the second phase is enhanced and more prolonged. This is termed as *hyperinsulinemia* due to the fact that higher time-integrated insulin release exists in such a pathological state, despite the first phase being inhibited. In healthy individuals, about 75% of the insulin-induced glucose uptake is ensured into skeletal muscle, whereas this is substantially reduced in hyperinsulinemic and obese patients (21).

With the progressing molecular physiology research, it is recognized that numerous other factors contribute to the fine blood glucose regulations and insulin responses, namely nutritional signaling mediated by the metabotropic receptors (122), endocrine role of incretins, that is, glucagon-like peptide 1 (GLP-1) (97, 158) and gastric inhibitory polypeptide (GIP) (198) (and other gastrointestinal hormones), paracrine GLP-1 signaling (82), paracrine and endocrine secretion of other hormones (245), systemic control by brain (43), and immune system contribution. Concerning the type 2 diabetes development, emphasis predominates mostly in relation to WAT on the so-called low-grade inflammation causing IR (20, 130, 308). During prediabetes, at early type 2 diabetes stages, a compensation phase exists when β-cells respond by enhancing their mass and function.

**FIG. 1.**
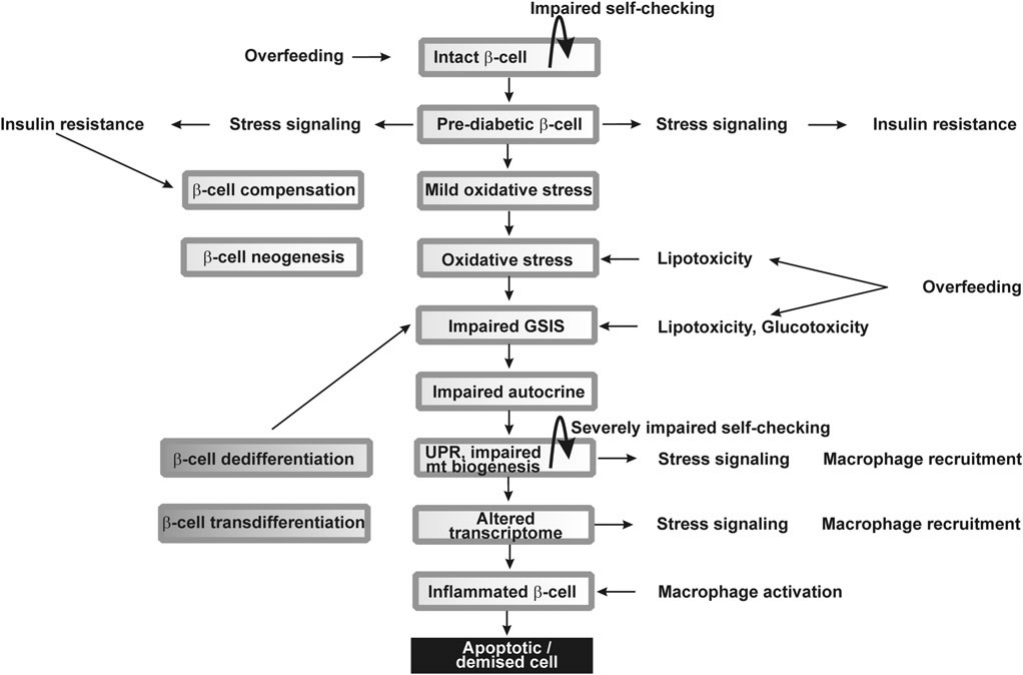
**Progress of type 2 diabetes development from the β-cells point of view.** Scheme shows a tentative sequence of events affecting pancreatic islet β-cells during type 2 diabetes progression. Hypothetical initial stimuli of overfeeding may shift the β-cell identity checking, thus transforming β-cells to a prediabetic state, in which they promote hypothetical stress signals causing partial insulin resistance of periphery besides the compensatory responses. The latter may include the excessive insulin expression leading to UPR and ER stress. The resulting mild oxidative stress accelerates into the intensive oxidative stress due to the subsequently ongoing lipotoxicity and glucotoxicity, while causing impaired GSIS and dysfunction to the other modes of insulin secretion. Resulting further turn of such a pathological spiral includes severely affected β-cell identity checking, causing the impaired *INS* gene expression, altered transcriptome, and β-cell dedifferentiation and transdifferentiation, which further deterioriate GSIS and the other modes of insulin secretion. At the final stage, systemic inflammation and islet inflammation can affect β-cell so to initiate apoptosis or other forms of cell death. ER, endoplasmic reticulum; GSIS, glucose-stimulated insulin secretion; UPR, unfolded protein response.

However, the overwhelming progress of such compensation induces further pathogenesis (7). Consequently, the onset of type 2 diabetes is accompanied by the inability of the existing functional β-cells to meet the altered, glucotoxic, metabolic demand (149) (Fig. 1).

In contrast, it is still a matter of debate whether the impaired autocrine factors such as autocrine insulin signaling participate in type 2 diabetes development. Two decades ago, the existence of the autocrine insulin secretion was considered plausible (14, 170), whereas it was later questioned and alternative explanations were elaborated explaining the apparent autocrine effects rather by the central nervous system regulation (241).

However, the impaired biogenesis and self-checking of pancreatic β-cells and islets, and specifically the loss of β-cell differentiated identity, are recently regarded as the most essential factors contributing to the origin of the type 2 diabetes (23, 270, 273), besides the low-grade inflammation in pancreatic islets, promoting β-cell oxidative stress, and cell death (Fig. 1). This is accompanied by a cross talk with the immune system and peripheral tissues. Nevertheless, this cross talk should be minimally substantiated by macrophage recruitment to the islets hypothetically initiated by the impaired “well-being” or self-checking and gradual loss of the β-cell identity. A common feature or outcome of some of these changes is either the oxidative stress or impaired redox signaling, and therefore, these aspects are reviewed here as the common theme.

A consensus exists for type 2 diabetes origin due to the overnutrition (252). The intensive overnutrition elevates the plasma levels of glucose and FAs, which cause metabolic stress at various organs and tissues including pancreatic β-cells. FAs and other metabolites act, for example, *via* numerous types of G-protein-coupled receptors (GPRs) family members (122). Sustained overnutrition not only progressively impairs the existing β-cells but also stimulates proliferation from their progenitors as a part of the adaptive responses. We should emphasize the compensatory role of β-cells in the initial phases of type 2 diabetes when increased β-cell biogenesis, and hence β-cell mass, elevates insulin gene expression attempting to compensate the insufficient insulin regulation. Despite the extensive description of this compensation, it is out of the scope of our review.

The involved phenomena of the increased antioxidant defense are within our scope. The compensation also involves a fine-tuned regulation to upgrade the endoplasmic reticulum (ER) capacity and quality control (237). Without such enhancement of ER capacity, the unfolded protein response (UPR) is initiated by the excessive unfolded proinsulin accumulation. All these changes include alternations of the identity checking and impaired “well-being” of β-cells.

Resulting tissue-specific metabolic stress thus also causes the release of cytokines and chemokines, which recruit immune cells and initiate the tissue-specific inflammation. Macrophages consist of a spectrum of cells with distinct functional plasticity. Among them, the two limiting classes are classified as M1 and M2 macrophages. One has to judge the M1/M2 macrophage proportion in diabetic pancreatic islets to discuss the molecular mechanism (77). Macrophages are activated by classical way, that is, M1 type are cytotoxic, produce superoxide (O_2_^•−^) by activated NADPH oxidases (NOXs) or HOCl and HSCN by myeloperoxidase into extracellular environment, and produce proinflammatory cytokines.

All these events may induce apoptotic or necrotic type of death in β-cells or may alter their redox regulations and expression of housekeeping and β-cell-identity maintaining genes. Theoretically, this may induce even β-cell dedifferentiation or transdifferentiation. In contrast, M2 macrophages activated by alternative way resolve inflammation, and the pancreatic islets reparation is provided by phagocytosis of apoptotic cells and rebuilding of the extracellular matrix (77). The M1/M2 macrophage proportion in diabetic pancreatic islets has not yet been satisfactorily evaluated. Nevertheless, obviously, the M1 macrophage excess should cause a sufficient deterioration of β-cells.

Undoubtedly, the IR development in peripheral tissues also stems predominantly from the inflammatory responses (107). Surprisingly, skeletal muscle-specific insulin receptor knockout mice exhibited IR but normal fasting glucose, hence it has been evidenced that IR stems from multiorgan deficiency, including that of β-cells (44). Peripheral tissue inflammation is connected with expression of cytokines, chemokines, and adipocytokines in WAT due to transcriptional activation of their expression by oxidative and metabolic stress. At the initial stages, we can consider them as a low-grade inflammation. The ER stress, by means of UPR, is another mechanism that plays a crucial role in the development of IR in adipocytes and peripheral tissues.

Recently, a redox nature of certain β-cell-identity checks and maintenance has been predicted, giving further importance to redox biology of pancreatic β-cells. Receiving the “wrong” identity checking signals might subsequently lead to a release of putative stress signals, the nature of which has yet to be determined. Such β-cell stress signals may hypothetically cause IR in peripheral tissues and may be combined or followed with inflammatory responses in pancreatic islets. Besides inflammatory signals, exosomes can also carry out the stress signals from pancreatic β-cells to peripheral tissues, such as transduced by microRNAs (miRNAs) (150). Such an emerging novel mechanism of IR induction has to be studied further.

#### Cross talk between stressed β-cells and IR

*Vice versa*, the IR affects back pancreatic β-cells, by several lines of mechanism. The ultimate consequence is the accelerated islet inflammation. However, metabolic components stemming from nearly permanent hyperinsulinemia and glycemia induce a further turn of additional oxidative stress through the actions of oxidated or proinflammatory lipid species, advanced glycation end (AGE) products, and amyloid peptide. Consequently, the only partial β-cell impairment, at early stages, later contributes to a further turn of pathology spiral leading to irreversible insufficiency in insulin secretion. One can recognize that the oxidative stress can be regarded as a common pathogenic factor in inflammation and metabolic effects of the evolving pathology.

Metabolic, hormonal, and immune-related pathological factors thus “fight back” to pancreatic β-cells and promote their insufficiencies, apoptosis, ER stress, impaired autocrine functions, identity checking signaling of “wrong identity,” and dysregulated biogenesis of remaining progenitor cells. A common factor here is the insufficient *INS* gene expression and impaired secretion of insulin as responding to normal physiological stimuli.

The major metabolic feedback is mediated by chronically elevated plasma glucose and FA levels, whereas the cytokine signaling at low-grade inflammation stimulates expression of the toll-like receptor 4 (TLR4) (33). All these events promote oxidative stress of β-cells, their ER stress, dysfunctional mitochondria, all of which, in higher progression, lead to β-cell apoptosis (255).

Some human populations were faced to pressure for survival in extremely cold climates, leading to the selection of combination of specific genes or alleles. As a result, these genes predisposed certain today's ethnic groups living in North Polar regions to the decreased prevalence of type 2 diabetes at the simultaneous enhancement of thermogenesis.

### Inflammation contribution to type 2 diabetes

#### Metabolic syndrome origin of diabetic complications

Obesity and lipodystrophy is substantiated by the abnormal mass of WAT, which in predominant cases results in the development of IR. Since WAT serves also as an endocrine organ regulating energy metabolism and insulin sensitivity by FAs and adipokines, its endocrine actions serve important homeostatic role. The adipokines include proinflammatory mediators such as leptin, tumor necrosis factor-α (TNF-α), interleukin (IL)-6, tissue inhibitor of metalloproteinases (TIMP-1), retinol-binding protein 4 (RBP-4), and monocyte chemotactic protein 1 (MCP-1), not only inducing local WAT inflammation but also systemic inflammation (215). The anti-inflammatory adiponectin, which declines in obesity, is also considered as an adipokine. Notably, an imbalance between leptin and adiponectin could also cause IR.

#### Origin of inflammatory responses

Proinflammatory cytokines contribute to IR development. Among them, interleukin-1β (IL-1β), IL-6, TNF-α, and the C-reactive protein (CRP) are taken as markers; however, the contribution of various other chemokines and adipocytokines may provide important responses. The master proinflammatory mediator is IL-1β since it induces production of other cytokines and chemokines. IL-1β binds to the interleukin-1 receptor type 1 (IL-1R1) and reduces the expression of insulin receptor substrate-1 (IRS-1) by the extracellular-related kinase- (ERK) dependent transcriptional regulation and also at post-transcriptional level (134). In this way, by reducing the ability to utilize insulin, the IR is developed in peripheral tissues.

IL-6 is known to be increased in plasma in aging. IL-6 activates the suppressor of cytokine signaling (SOCS) proteins blocking the signal transducer and activator of transcription 5B (STAT5B), a transcription factor of insulin receptor. As a result, IL-6 contributes to the IR development. Moreover, IL-6 suppresses the lipoprotein lipase, which leads to the increasing plasma levels of triglycerides (159). TNF-α, produced also by adipocytes, activates cascades of the nuclear factor kappa-light-chain-enhancer of activated B cells (NF-κB) and c-Jun N-terminal kinase (JNK), phosphorylating serine 307 in insulin receptor substrate IRS-1, thus contributing to IR (2).

Chemokines include, for example, MCP-1, MCP-2, MCP-3, MCP-4, CCL2, MIP-1α, and MIP-1β (224). Their involvement in IR development is nicely illustrated by the inability of high-fat diet to induce IR in MCP-1- and CCL2-deficient mice (152, 296). Chemokine receptors such as CCR2 and CCR5 in WAT are important for IR development. Besides inducing expression of other inflammatory genes, the activation of CCR2 inhibits insulin-dependent glucose uptake into adipocytes.

#### Pancreatic islet inflammation involving β-cells

Pancreatic islet inflammation is caused by the interaction of M1-like macrophages and β-cells producing inflammatory cytokines in vicious cycle. This, of course, predominates in type 1 diabetes. The number of macrophages increases in Langerhans islets during type 2 diabetes, thus elevating the production of proinflammatory cytokines (79). Macrophages also produce chemokines, which are essential for the recruitment of innate immune cells into pancreas along with promoting changes in signaling of resident leucocytes (59). It has been documented that M1 type of macrophages infiltration into islets of pancreas can induce a significant loss of β-cells (169).

Such an example of NLRP3 inflammasome activation (the third isoform of NLRP [NOD-like receptor containing pyrin domain]) in infiltrating macrophages is endocannabinoids, which contribute to lowering of β-cell mass in type 2 diabetes (144, 145). Classical activation of M1 macrophages induces inflammation-dependent superoxide and HOCl or HSCN and contributes to already existing oxidated stress in β-cells. Produced proinflammatory cytokines, such as IL-1β, clearly enhance the number of apoptotic pancreatic β-cells, manifested as cytochrome *c* release from mitochondria followed by downstream activation of caspases (56). Increased reactive oxygen species (ROS) production and subsequent redox regulations were reportedly shown as mediators of the cytokine-induced cell death since overexpression of antioxidant enzymes prevented this process (16). Cytokines were also found to induce ER stress (42).

IL-1β induces inflammation in islets due to glucolipotoxicity, that is, prolonged exposure to FAs, glucose, or other nutrients. FAs seem to play the role of a main regulator of inflammation in β-cells. In β-cells, FAs act *via* GPRs, *via* the TLR2,4/MYD88/NF-κB pathway (where MYD88 [myeloid differentiation primary response 88 protein] is involved), and *via* the NLRP3 inflammasome/apoptosis-associated speck-like protein containing CARD (ASC)/caspase 1 pathway to increase IL-1β expression (312). Here, ASC is the ASC.

Glucose stimulates IL-1β expression by the thioredoxin-interacting protein (TXNIP) upstream of the NLRP3 inflammasome. Resulting IL-1β exerts an autocrine function by the activation of its receptor IL-1RI recruiting MYD88 and accelerating its own expression, whereas the activation of MYD88/NF-κB pathway stimulates the expression of CCL2, CCL3, and CXCL8 chemokines. The chemokines recruit macrophages also producing IL-1β (78). In later diabetes stages, islet amyloid stimulates inflammasome in the islet-recruited macrophages. Excessive palmitate was also found to mediate TXNIP downregulation (225).

Excess of saturated FAs (*e.g*., palmitate) induce chemokine and cytokine expression (CXCL1, CCL3, IL-6, and IL-8) within Langerhans islets *in vitro* (56, 123). Human islet donors with type 2 diabetes were shown to induce transcription of cytokine and chemokine (193, 274). Moreover, analyses of laser microdissected β-cells from type 2 diabetic donors have shown induced expression of cytokines IL-1β, IL-8, and IL-11, and chemokines CCL2, CCL11, and CCL13, plus downregulation of IL-1α cytokine (195). Notably, nondiabetic control samples have not uncovered IL-1β presence by *in situ* hybridization (34).

The S100 calcium-binding protein A8 (S100A8), a member of the damage-associated molecular pattern molecules, has been implicated in β-cell inflammation (128). Its expression was increased by TLR4 signaling in pancreatic islets induced by palmitate in the presence of a high glucose during their co-culturing with unstimulated peritoneal macrophages. S100A8 was suggested to mediate islet–macrophages interaction that results in β-cell apoptosis. TLR4 pathway inhibition avoided this interaction. Also, Fetuin-A secretion induced by palmitate acts *via* the NF-κB/MIF inflammatory pathway (MIF is the macrophage migration inhibitory factor). Fetuin-A is an α-2-HS-glycoprotein excreted by the liver. In pancreatic islets, Fetuin-A secretion sets “an inflammatory environment” leading to β-cell dysfunction (209).

Lipotoxicity can be ensured by major lipoxygenase in human pancreatic islets, such as by 12-LO-producing 12-S hydroxyeicosatetraenoic acid (12-S HETE) from arachidonic acid in β-cells. Elevated 12-S-HETE was found in islets and WAT of rodent diabetes models and diabetic patients. Proinflammatory 12-S-HETE induces cytokine production, such as IL-12, and activates JNK and oxidative stress pathways by p38 mitogen-activated protein kinase (MAPK), which induces the NOX1 activity. Consequently, the 12-S-HETE effects lead to the decreased β-cell viability and impaired insulin secretion (125).

## Oxidative Stress and Antioxidant Protection in Pancreatic β-Cell

### Damages to perfect glucose sensor

#### Glucose sensing in pancreatic β-cells

The current consensus on the glucose-sensing function of pancreatic β-cells lies in the increasing oxidative phosphorylation (OXPHOS) rate with the elevated glucose metabolism of incoming glucose (13, 164, 231, 246, 299). The resulting elevated ATP/ADP ratio closes the plasma membrane ATP-sensitive K^+^ channel (K_ATP_), which then initiates orchestra of other channel events substantiating plasma membrane depolarization (72). This activates voltage-gated L-type Ca^2+^channels (Ca_L_) leading to Ca^2+^ entry and hence Ca^2+^-dependent exocytosis of the insulin-containing secretory granules.

**FIG. 2.**
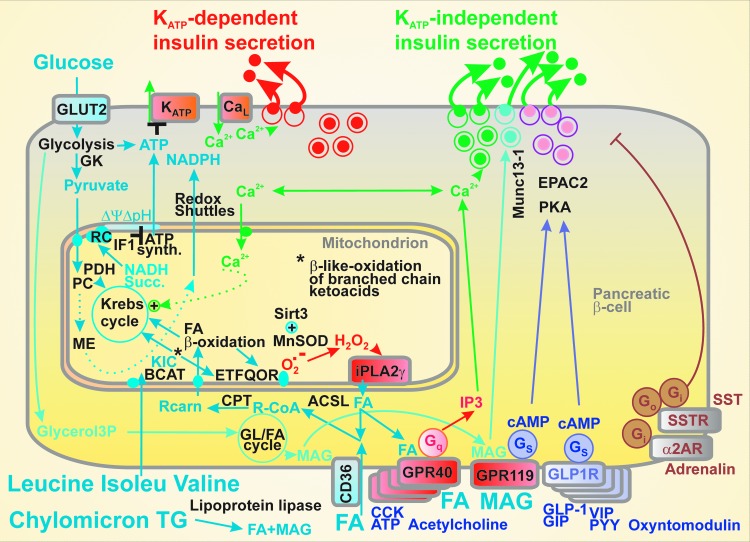
**Mechanisms of insulin secretion.** Emphasizing the K_ATP_-dependent and K_ATP_-independent pathways, which are interrelated namely by Ca^2+^ homeostasis, the scheme illustrates major metabolic and receptor-mediated pathways of insulin secretion, including the inhibitory pathways of α2-adrenergic and somatostatin (SST) receptors (α2AR, SSTR), respectively. GPRs for amino acids were not illustrated for simplicity. ASCL, long-chain acyl-CoA synthetases; BCAT, branched chain ketoacid aminotransferase; CCK, cholesystokinin; CPT, carnitine palmitoyl transferase; FA, (free) fatty acids; GK, glucokinase; GLUT2, glucose transporter 2; GPRs, G-protein-coupled receptors; K_ATP_, ATP-sensitive K^+^ channel; KIC, 2-ketoisocaproate; MAG, monoacylglycerol; PC, pyruvate carboxylase; PKA, protein kinase A; PYY, peptide Y; RC, respiratory chain; VIP, vasoactive intestinal peptide.

A large facilitation of insulin secretion exists due to the intestinal incretin hormone action. This includes GLP-1 stimulation *via* the GLP-1 receptor, Gs protein, adenylate kinase activation, and cAMP-dependent activation of the protein kinase A (PKA), and the exchange protein directly activated by cAMP 2 (EPAC2) (97). Both downstream pathways promote Ca^2+^-dependent exocytosis of insulin granules independent of K_ATP_ (Fig. 2). A similar stimulation exists with GIP (198).

In addition, acetylcholine stimulates insulin secretion *via* muscarinic M3 receptors, extracellular ATP stimulates *via* purinoreceptors (246), and long-chain FAs *via* metabotropic receptors GPR40 (98, 113, 122, 129, 194). Activation of all these receptors is followed by the Gq pathway promoting the inositol trisphosphate- (IP3) mediated Ca^2+^ release, again independent of K_ATP_. Other metabolites, such as aromatic amino acids, act *via* distinct GPRs (122). Also, the glycerol/FA cycle relevant for 2-monoacyl glycerol-mediated insulin secretion acts independent of K_ATP_ *via* Munc13-1 facilitation of exocytosis (231). Notably, insulin release can be inhibited by acting *via* the inhibitory Gi proteins upon the activation of α2-adrenergic receptors or somatostatin receptors *via* the inhibitory Gi and Go proteins (246).

Nature's engineering of the glucose sensor consists of several key points, without which the sensing mechanism would never work. At first, glucose transporter GLUT2 (or probably GLUT1 in humans) is constitutively active and is not dependent on the insulin receptor signaling cascade as, for example, in peripheral tissues. This allows glucose concentration in the β-cell cytosol being proportional to bloodstream glucose (151).

Second, the suppressed expression of lactate dehydrogenase (belonging to the so-called β-cell-disallowed genes) and the pattern of pyruvate dehydrogenase kinase (*Pdk*) genes are surely responsible for the fact that all glucose is metabolized by OXPHOS in β-cells.

Thus, β-cell PDK1 (pyruvate dehydrogenase kinase 1) and PDK3 are “constitutively blocked” (5), and PDK2 is “inefficient” so that it does not phosphorylate the E1α subunit of pyruvate dehydrogenase (PDH), hence does not inhibit its activity. At low basal glucose, PDH shows high activity, whereas at increased glucose, PDH is inhibited only by 22%.

The third aspect includes the fact that hexokinase IV (glucokinase) in β-cells is not inhibited by glucose-6-phosphate like other hexokinase isoforms (226). The missing feedback inhibition of glycolysis directly connects glycolysis to pyruvate. Consequently, the β-cell respiration and OXPHOS rates, leading to increased ATP/ADP ratio, are dependent on glucose availability, whereas in most other cell types, cell demand dictates respiration/metabolism rates and the ATP/ADP ratio.

Fourth, matching of physiological postprandial glucose concentrations with the sensitivity range of the glucose sensor (which pancreatic β-cells indeed represent) is ensured by the inhibitory factor IF1 of the mitochondrial ATP synthase (69). This aspect was derived from the IF1 ablation experiments, which allowed switching on the sensor, that is, elevation of respiration and OXPHOS, even at zero glucose concentration and shifted the insulin release dose–response to the range of 0–2 m*M* glucose. This demonstrated that IF1 ensures that mitochondrial respiration and resulting ATP synthesis and insulin release starts to increase sharply at ∼3 m*M* glucose levels due to a weak inhibition of the ATP synthase (and hence OXPHOS) by IF1.

Consequently, glucose metabolism in β-cells is finely adjusted to the blood glucose levels (201). Also, the above-mentioned complete repression of high-affinity type I–III hexokinases contributes to the prevention of insulin release at low plasma glucose concentrations (254).

At starvation with approximately 3–5 m*M* glucose levels, β-cell respiration as well as the intensity of ATP synthesis are low (263). The increase in glucose concentrations raises OXPHOS up to saturated concentration at approximately 12–15 m*M* glucose, when cells show maximum OXPHOS, maximum respiration and mitochondrial inner membrane potential ΔΨ_m_.

Another peculiar aspect of the pancreatic β-cell glucose sensor dose–response, that is, glucose dependence of insulin response lies in its steepness, higher than would correspond to a Hill coefficient of 1. We have revealed that upon glucose-stimulated insulin secretion (GSIS), mitochondrial cristae become narrowed at glucose intake in model β-cells and, consequently, proton coupling localized into smaller intracristal space volume enables more efficient OXPHOS, thus contributing to the steepness of the glucose sensor (148). Overall, β-cells maintain a relatively high [ATP]/[ADP] value even in low glucose and glucose metabolism dramatically decreases free ADP while only modestly increases ATP (88). Since a high [ATP]/[ADP] ratio exists even at low glucose levels, the total adenine nucleotide content is unchanged during a glucose-induced elevation.

After a meal intake, blood glucose rises slowly and insulin release appears to be monophasic. Experimentally after a bolus of glucose, insulin release proceeds in two phases (287), which are also observed in perifusion experiments of the isolated pancreatic islets. Earlier considerations regarded the first phase as purely K_ATP_-dependent, whereas the second phase was considered to occur due to the K_ATP_-independent mechanism. However, this concept turned out to be incorrect since both classes of mechanisms contribute to both phases (13, 114, 231, 246).

Actually, Ca^2+^ elevations during GSIS have two phases, a sharp increase after glucose intake, which is followed by the prolonged oscillations (4). The first GSIS phase was also considered to be executed by a readily releasable pool of granules docked to the plasma membrane, whereas the second phase by a reserve pool of granules (13, 231, 246). Note that the insulin-degrading enzyme, an endopeptidase, was found in β-cells and α-cells (83), degrading insulin and glucagon. This should be the subject of further studies, whether it may also contribute to the phases, beside the insulin receptor internalization (see the Autocrine Insulin section).

#### Relationships between ROS homeostasis and glucose sensor

Redox homeostasis in cells and particularly in mitochondria and peroxisomes is physiologically regulated in multiple hierarchies and in pancreatic β-cells contributes to the mechanism(s) of insulin secretion. When these physiological relationships are dysregulated, an internal oxidative stress in pancreatic β-cells arises, which inhibits insulin secretion (178, 216). At the organism and islet levels, inflammation amends an additional component of the oxidative stress in β-cells.

**FIG. 3.**
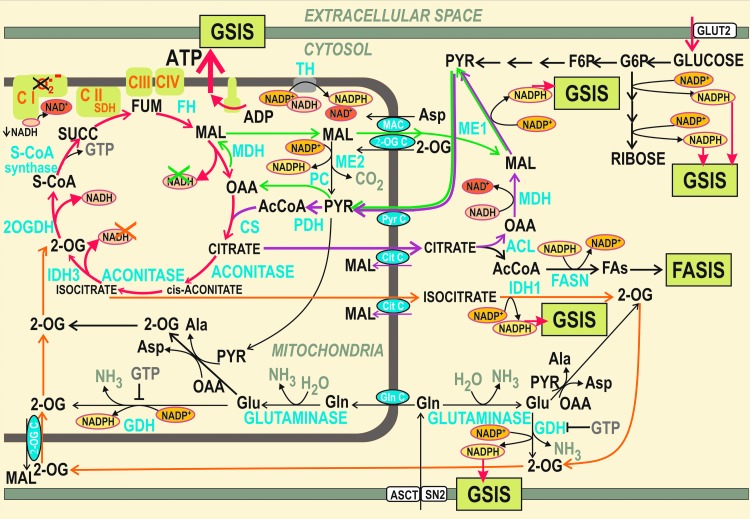
**NADPH facilitation of GSIS and mitochondrial redox state.** Mitochondrial redox shuttles exporting reducing equivalents from the mitochondrial matrix to the cytosol are illustrated. The resulting elevated cytosolic NADPH has been reported to facilitate GSIS. The shuttles are depicted by *arrows* of distinct colors: *dark green*: pyruvate/malate shuttle; *purple*: pyruvate/citrate shuttle; and *orange*: pyruvate/isocitrate shuttle. When functional at the glucose intake, for example, the pyruvate/malate shuttle is enabled by the pyruvate carboxylase reaction; NADPH is elevated in the cytosol at the expense of unmade matrix NADH. Consequently, a lower NADH/NAD^+^ ratio is predicted to cause diminished superoxide formation within the Complex I, and hence, functional GSIS hypothetically should not increase matrix oxidative stress. At impaired GSIS and exaggerated metabolism, resulting higher NADH/NAD^+^ ratio should then increase superoxide formation and hence the matrix oxidative stress. 2-OG, 2-oxoglutarate; 2-OG C, 2-oxoglutarate carrier; 2OGDH, 2-oxoglutarate dehydrogenase; ACL, ATP citrate lyase; Cit C, citrate carrier; CS, citrate synthase; FASIS, fatty acid-stimulated insulin secretion; FASN, fatty acid synthase; FH, fumarate hydratase; GDH, glutamate dehydrogenase; MAC, malate aspartate carrier; MDH, malate dehydrogenase; ME, malic enzyme; Pyr C, pyruvate carrier; S-CoA, succinyl coenzyme A; SDH, succinate dehydrogenase.

Under physiological situation, GSIS in pancreatic β-cells is facilitated by the increase in cytosolic NADPH (13, 141, 143, 219, 231, 244, 246) or even by ROS (176, 228), such as resulting from the addition of mono-oleoyl-glycerol (247). The three major metabolic shuttles virtually export the reducing equivalents of NADH from the mitochondrial matrix to the cytosol during GSIS (141). They were named as the pyruvate/malate, pyruvate/citrate, and pyruvate/isocitrate shuttle (143). The two cytosolic enzymes produce NADPH, the cytosolic malic enzyme 1 (ME1) within the first two shuttles, whereas the isocitrate dehydrogenase 1 (IDH1) acts within the third one (141). As a result, the GSIS is facilitated by the NADPH increase, a mechanism that has yet to be revealed (Fig. 3).

These redox shuttles also affect ROS homeostasis. For each molecule of malate or citrate/isocitrate exported from the mitochondrial matrix by the respective shuttle, one molecule of NADH is missing for the respiratory chain Complex II since the malate dehydrogenase (MDH) or IDH3, otherwise producing NADH, cannot use their substrates. Malate can be also cycled back after being converted to pyruvate by mitochondrial ME2. Consequently, the matrix NADH formation is predicted to be lower and NAD^+^ is assumed to accumulate in the matrix upon GSIS, specifically when compared with the situation when these shuttles are slowed down or blocked.

At elevated glucose metabolism, these shuttles exporting redox equivalents should hypothetically decrease the substrate pressure NADH/NAD^+^ in the mitochondrial matrix, which would lead to the decreased superoxide formation in the flavin site I_F_ of Complex I. Overall, this hypothetical mechanism would contribute to the diminished superoxide formation upon GSIS and should effectively prevent the ROS increase upon GSIS within mitochondria (Fig. 3).

Yet, previously, it has been reported that mitochondrial superoxide formation increases upon GSIS (32, 176, 228). In contrast, it has been found that low glucose levels induce ROS production in pancreatic β-cells (118, 251) and that ROS were suppressed by high glucose (196). The glucose decrease was also associated with the increased mitochondrial H_2_O_2_ (243).

Also, channel machinery has been related to ROS. Insulin granule exocytosis has been reported to be directly induced by H_2_O_2_, independent of Ca^2+^ (189). Plasma membrane depolarization may be hypothetically affected by direct or indirect inhibition of K^+^_ATP_ channel by H_2_O_2_ (305) or by direct or indirect inhibition of repolarizing K^+^ channels, such as K_V_ (11, 87, 181, 190, 202, 303) or activation of TPRM2 (transient receptor potential melastatin 2) depolarizing channel (101). Indirect blockage would involve ROS-activated signaling pathways leading to K_ATP_ or K_V_ closure. Also, the Ca^2+^-mobilizing messenger nicotinic adenine nucleotide diphosphate (NAADP) acts *via* endosomal and lysosomal two-pore channels (TPC) (12).

#### Physiological antioxidant mechanism in pancreatic β-cells

Next, let us discuss about the mechanisms that can be manifested in maintaining the physiological ROS homeostasis and their impaired shift to oxidative stress under pathological conditions. Nature engineered a plethora of native mechanisms to suppress the superoxide sources within the mitochondrial respiratory chain.

Recently, one of these mechanisms was found to be executed by the synergy of mitochondrial-specific calcium-independent phospholipase A2, isoform γ (iPLA2γ), and uncoupling protein UCP2 (136). Uncoupling cannot suppress superoxide formation when arising from the retarded electron transport pathway and proton pumps by mutations of subunits encoded by mitochondrial DNA (mtDNA) or when superoxide is formed due to the retarded cytochrome c shuttling (229). Otherwise, uncoupling by UCPs (UCP1–UCP5) is quite an effective way to suppress mitochondrial superoxide formation (138, 180).

Sole respiration and OXPHOS may increase mitochondrial formation of superoxide when a certain redox reaction is retarded and the input metabolic (*e.g*., NADH) flux keeps a metabolic pressure. Excessive superoxide is subsequently dismuted by the matrix-located SOD2/MnSOD. At sufficient H_2_O_2_ degradation, MnSOD thus has profound antioxidant effects. This substantiates the beneficial effects of Mn supplementation (174). MnSOD can be prooxidant when downstream degradation of H_2_O_2_ does not match its formation.

However, MnSOD can be inactivated by acetylation that is, in turn, rescued by the sirtuin-3-mediated deacetylation (67, 275, 276). Sirtuins in general promote an OXPHOS and OXPHOS-supporting pathways, affecting the involved proteins. Acetylation of numerous mitochondrial proteins inhibits their function, whereas their deacetylation by certain sirtuins abolishes such inhibition. Among mitochondrial sirtuins, sirtuin 3 is an NAD^+^-dependent deacetylase essential for OXPHOS (50, 160, 267, 313), whereas sirtuin 5 is a potent desuccinylase (309). Among others, subunits of the ATP synthase, OPA1, or MnSOD are regulated by the inhibitory acetylation and physiologically activated by sirtuin 3 (55), whereas enzymes of FA β-oxidation and ketogenesis are activated by desuccinylation mediated by sirtuin 5 (238). Mitochondrial sirtuin 4 removes three acyl moieties from lysine residues (9).

Numerous cases were found when sirtuin function has been linked to insulin secretion. Also, typically, sirtuin 3 expression is reduced upon high-fat diet suggesting the involvement of the oxidative stress arising from partly inactive MnSOD (313).

When H_2_O_2_ is formed, it diffuses out of mitochondria or from peroxisomes into the cytosol. Organelles and cytosolic milieu contain redox buffers and redox relaying systems providing redox signal propagation, besides a simple maintenance of the redox homeostasis. Thus, disulfide reductases, thioredoxins (TRXs), glutaredoxins (GRXs), peroxiredoxins (PRXs), and glutamate-cysteine ligase are also capable to propagate the redox signals to the targets.

TRX are disulfide reductases of protein sulfhydryl groups, maintaining proteins in the reduced state (17). The oxidized TRX are regenerated by the TRX reductase using NADPH for electrons. Similarly, GRX reductase-2 reduces H_2_O_2_ or hydroperoxy-FA lipid chains to water or hydroxy FA lipid chains, respectively, on the expense of conversion of reduced glutathione (GSH) to oxidized glutathione (GSSG) (240). Glutathione is regenerated by glutathione reductase.

PRX, as thiol peroxide reductases, use TRX or other thiol-containing proteins to clear H_2_O_2_ or lipid peroxides (311). The catalytic cysteine sulfhydryl group of PRXs is selectively oxidized by H_2_O_2_ to either sulfenic acid or disulfide intermediates. At the TRX shortage, PRX is inactivated to PRX-SO2, which can be reversed by sulfiredoxins, at the expense of ATP, yielding sulfenylated PRX (PRX-SOH) (302). PRX are classified into two major enzyme classes, differing by the mechanism of recycling of the sulfenic acid back to a thiol. 2-Cys PRXs are reduced by thiols such as of TRX, TRX-like proteins, or in certain cases by glutathione. The 1-Cys class of PRXs is reduced by ascorbic acid or glutathione in the presence of GST-π (206). Note that PRX3 is localized to mitochondria, where besides redox buffer function exerts its role in the redox H_2_O_2_ signaling.

#### Oxidative damage *versus* antioxidant defense in pancreatic β-cells

Oxidative stress is induced when redox homeostasis diverts to excessive ROS production, which exceeds the antioxidant mechanisms. A general oxidative stress in a cell arises when the function of redox buffers and antioxidant enzymes is diminished so that they no longer possess the ability to detoxify the produced ROS. A permanent character and the level of ROS amounts distinguish this stress from the physiological redox signaling. Oxidative stress possesses not only the direct pathological consequences, by increased appearance of oxidatively modified biological constituents, but also the induction novel cellular responses, that is, programmed cell death, such as apoptosis. Usually, these phenomena occur in parallel, after accumulation of the oxidative stress reached a certain threshold.

Oxidative stress can oxidatively modify DNA with more vulnerable mtDNA, lipids (by nonenzymatic lipid peroxidation), and proteins, such as carbonylation. Although cell owes physiological mechanisms for clearance of these oxidized constituents (repair mechanisms for DNA, glutathione peroxidase [GPX], and normal protein turnover), when their formation exceeds clearance capacity, some pathology can occur. Yet imbalance in identity check mechanisms in a cell can induce such stress even at mild oxidative stress. In this case, an impairment of normal autophagy and notably autophagic mechanisms dealing with mitochondria leads to the accumulation of products that were supposed to be cleared with serious consequences for the cell (157, 293).

A hallmark of β-cell ROS homeostasis is that redox buffers and antioxidant enzymes are rather weak when compared with other cell types (177), and consequently, the ROS homeostasis can be easily shifted to the oxidative stress conditions. Catalase, GPX, and superoxide dismutase (SOD1 or CuZnSOD) represent three of most important intracellular antioxidant enzymes of a primary defense system. However, pancreatic islets contain only 1% catalase, 2% GPX, and 29% SOD1 activities compared with the liver (100, 179, 203, 280). This makes them more susceptible to an oxidative insult. Human β-cells were suggested to be less prone to oxidative stress than rodent β-cells, possibly because they show enhanced catalase and SOD activity (297). But GPX activity is poorly detectable in human β-cells (281). Moreover, β-cells possess weak repair machinery for oxidatively damaged DNA (204). Besides small antioxidant molecules, that is, vitamin E (α-tocopherol), ascorbate, or uric acid, glutathione provides an important antioxidant protection of the β-cells against oxidative damage (167, 211). Glutathione, in millimolar concentrations, is kept in the reduced state (GSH) by glutathione reductase (272). GSH transfers its reducing equivalents to ascorbate, GPX, and GRX.

In turn, β-cells are rich in disulfide reductase-based antioxidant defenses, such as GRX and TRX (131). They were shown to be localized at periplasma membrane cytosol, and glutaredoxin GRX1 has been found to be implicated in the modulation of Ca^2+^-dependent insulin exocytosis [143]. NADPH was shown to stimulate the exocytotic machinery, which correlates with ∼30% inhibition in whole-cell Ca^2+^ currents. When GRX1 was silenced, NADPH was not amplifier of insulin release but still inhibited Ca^2+^ currents (240).

Although oxidative stress and metabolic dysregulation are considered as two of the most important pathogenic factors of impaired insulin secretion, the latter is usually regarded as dysregulated relationships between oxidation of glucose and FAs. Yet, there is still no consensus on what particular oxidants are responsive for oxidative stress and what exactly causes such a metabolic dysregulation. One type of the frequently overlooked products of oxidative stress is isoprostanes, which are prostaglandin-like compounds produced by nonenzymatic peroxidation of arachidonic acid (such as initiated by the hydroperoxyl radical, a conjugated acid of superoxide), together with a class of extremely reactive molecules that are generated as products of the isoprostane pathway (53). The levulgandins and isolevuglandins (currently referred to as isoketals) have been found to form covalent adducts with proteins and induce protein–protein and DNA–protein cross-links and thus are able to modify key biomolecules that are critical to normal cellular function and replication (249a). The levulgandins and isolevuglandins can accumulate over the lifetimes of proteins, and isolevuglandin–protein adducts represent a convenient dosimeter of oxidative stress central to many disease states (249a).

Unfortunately, despite increasing number of studies, which relate products of lipid peroxidation to the etiology of diabetes mellitus, the specific role of the isoprostane pathway of autoxidation of polyunsaturated fatty acids (PUFAs) in the regulation of insulin secretion has not been identified.

#### Kelch-like ECH-associated protein 1–nuclear factor erythroid 2-related factor system

Cells possess a protection against oxidative, electrophilic, or environmental stresses by activating genes *via* antioxidant-responsive elements facilitating the defense. Such a protective system includes the nuclear factor erythroid 2-related factor (Nrf2) together with a sensor Kelch-like ECH-associated protein 1 (Keap1). Nrf2 belongs to a family of transcriptional factors containing a unique basic-leucine-zipper motif, the cap-n-collar family [reviewed in Uruno *et al*. (284)]. Keap1 plays a substrate adaptor function and subjects Nrf2 to ubiquitination and degradation and thus suppress the transcriptional activity of Nrf2 under conditions of no oxidative stress. When cells are exposed to increased prooxidant conditions and oxidative stress, the cysteine residues of Keap1 are modified and Keap1 loses its ability to ubiquitinate Nrf2. Nrf2 then accumulates within the cell and induces the expression of target genes.

Nrf2-knockout islets and Nrf2-knockdown MIN6 cells were used to study the protective role of Nrf2 against acute oxidative stress-induced pancreatic β-cell damage (89). Both cells and islets expressed markedly lower amount of antioxidant enzymes in response to a variety of oxidative stressors, whereas pretreatment of β-cells with Nrf2 activators induced protection of the cells from H_2_O_2_-induced cell damage. Because it was shown that oxidants can amplify GSIS and persistent activation of Nrf2 decreases glucose-triggered ROS signaling insulin release, this study points out on distinct roles that Nrf2 may play in pancreatic β-cell dysfunction that accompanies different stages of diabetes.

The association of Nrf2 and inflammatory cytokines was found by a cross-sectional study involving healthy control subjects and subjects with newly diagnosed diabetes mellitus. Thus, while oxidative stress markers were significantly increased, the Nrf2 activity and its downstream targets were decreased in peripheral blood mononuclear cells of diabetic subjects in comparison with control. The circulatory increased levels of Nrf2 showed a positive correlation with anti-inflammatory Th2 type cytokines and negative correlation with proinflammatory Th1 type cytokines. Furthermore, the activation of Nrf2 restored the cytokine stress-induced impaired insulin secretion in pancreatic β-cells (259).

Interestingly, Nrf2 nuclear translocation was elevated in embryonic development in UCP2 knockout mice, which revealed increased phosphorylation of protein kinase B (Akt) in their pancreata, suggesting that UCP2 controls pancreas development through ROS-Akt signaling (41). UCP2 was shown to mediate its antioxidant function by a synergy with mitochondrial phospholipase iPLA2γ (133), which leads to the prevention of lipotoxic oxidative stress in pancreatic β-cells (136), indicating that the oxidative stress observed in the pancreas by the ablation of *Ucp2* gene is caused by the lack of iPLA2γ-UCP2 antioxidant synergy.

In addition, Nrf2 plays a role in the molecular mechanism of antioxidative effect induced by GLP-1. Results obtained with rat insulinoma cell line INS-1E suggest that exendin-4, a GLP-1 receptor agonist, activates and stabilizes Nrf2 through PKCδ activation, thus leading to the elevation of antioxidant gene expression, which then improves β-cell function during oxidative stress (161). GLP-1 activation of Nrf2 pathway with subsequent increase of antioxidant capacity signals *via* PKA-dependent ERK pathway, which induces Nrf2 nuclear translocation (84).

A chemokine C-X-C ligand 12 enhances survival and regeneration of pancreatic β-cells (217) and was shown to exert an antioxidant protection also *via* Nrf2 nuclear translocation through the activation of p38, ERK, and Akt kinases downstream of the chemokine receptor, thus contributing to the functional resistance of β-cells to prooxidant-induced stress (68).

#### Conditions for elevated ROS in pancreatic β-cells

Let us recapitulate the major events elevating ROS in β-cells. Mitochondria are traditionally reported as a profound source of oxidative stress in β-cell pathology (216). This can be deduced also from the effects of mitochondrial matrix-targeted antioxidants (126, 182).

As mentioned above, mitochondria may exhibit oxidative stress even in fasting conditions with low glucose levels (251). Mitochondrial respiration, especially when retarded at certain sites, provides the most upstream ROS, superoxide and its conjugated acid—hydroperoxyl radical, HO_2_^•^ (pKa 4.9) (137, 229, 268, 286). Superoxide is converted to H_2_O_2_ by SOD, cytosolic and intracristal space SOD1/ZnCuSOD (221, 291), and matrix SOD2/MnSOD. H_2_O_2_ may be converted by Fenton reaction in the presence of iron to the most reactive species—to the hydroxyl radical, ^•^OH, acting locally. The hydroperoxyl radical, HO_2_^•^, and hydroxyl radical, ^•^OH, are capable to initiate nonenzymatic lipid peroxidation, a further amplifier of ROS (197).

Resulting hydroperoxy FAs, hydroxy FAs (converted from hydroperoxy FAs by GPX4), and numerous other derivatives of PUFAs coming from enzymatic lipid peroxidation are cleaved from oxidized lipids by phospholipases A2 (261). More frequently, the shorter lipid peroxidation products, for example, arachidonic acid metabolites act as proinflammatory, whereas resolvins coming from C22 ω-3 polyunsaturated fatty acids (PUFAs) act as anti-inflammatory (256).

Reports emphasized the function of succinate dehydrogenase as another ROS source (76). Other superoxide source is related to mitochondrial FA β-oxidation, when superoxide is produced at the E_F_ site of electron-transferring flavoprotein:Q oxidoreductase (ETF:QOR) system (36). An ultimate MnSOD reaction in the matrix dismutes superoxide to H_2_O_2_, which activates iPLA2γ (136). Under conditions when concomitant activation of antioxidant synergy of UCP2 and iPLA2γ does not protect against these elevated, lipotoxic oxidative stress may arise (see the FA Sensing in Pancreatic β-Cells—Intact *Versus* Impaired section).

Cytosolic ROS are produced in pancreatic β-cells in association with the elevation of Ca^2+^ following the activation of protein kinase C (PKC), which promotes the assembly of and superoxide formation by NOXs (307a, 207).

#### Consequences of oxidative stress for glucose sensor

**FIG. 4.**
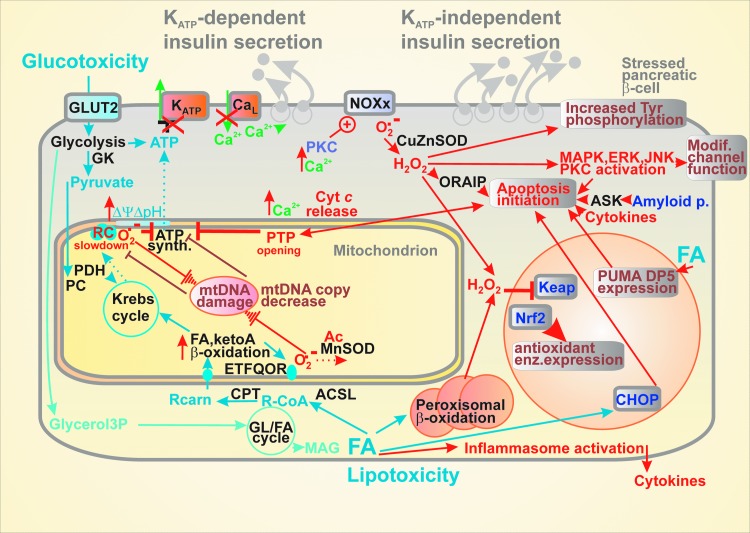
**Selected consequences of glucotoxicity, lipotoxicity, and islet inflammation for β-cell.** Glucotoxicity represented by the elevated glucose intake (besides AGEs) promotes increased mitochondrial superoxide formation, mtDNA damage, which together with other insults lead to the decreased or eliminated ATP synthesis responses on glucose metabolism, hence the impaired glucose sensor and consequently GSIS. Besides its inflammatory branch, lipotoxicity, represented by increased FA and MAG intake, causes disbalance in glycerol/FA cycle, increased mitochondrial superoxide formation, causing again mtDNA damage. Peroxisomes are important accelerators of lipotoxicity toward elevated H_2_O_2_ in the cytosol. After overriding the Nrf2-mediated antioxidant protection, the concerted mitochondrial and cytosolic oxidative stress and dysregulation of Ca^2+^ homeostasis initiate mitochondrially induced apoptosis and activate assembly of inducible NOX enzymes (all isoforms except NOX4). Apoptosis can be also initiated *via* the CHOP or PUMA pathways. Also, H_2_O_2_-regulated kinases switch their targets toward the proapoptotic regimen, modify function of various channels, further deteriorating the K_ATP_-branch of insulin secretion. AGE, advanced glycation end; CHOP, CCAAT-enhancer binding protein homolog; mtDNA, mitochondrial DNA; NOX, NADPH oxidase; Nrf2, nuclear factor erythroid 2-related factor; PUMA, p53-upregulated modulator of apoptosis.

Let us discuss the consequences of oxidative stress for glucose sensing in pancreatic β-cells except for the stress given by FAs, which is described below in the FA Sensing in Pancreatic β-Cells—Intact *Versus* Impaired section (289). At first, the oxidative stress can affect mitochondria with the ultimate consequences manifested in diminished ATP synthesis by OXPHOS and thereby impairment of the K_ATP_-dependent glucose sensing (85, 157, 191) (Fig. 4).

Mitochondrial dysfunction may result in induction of compensatory mechanisms such as the mitochondrial UPR, mitophagy, and autophagy. These systems, however, can be overcome by concomitant apoptosis and consequently reducing mass of β-cells and hence impairing insulin secretion and initiating the diabetic pathology. Nevertheless, the importance of autophagy can be illustrated by the effect of intermittent fasting, which preserves organelle quality *via* the autophagy–lysosome pathway and increases β-cell survival by stimulation of regeneration in obesity-induced diabetes (185). Thyroid hormones are also essential for β-cell maintenance as reflected by GSIS suppression, decrease in OXPHOS, and glucokinase activity in hypothyroid rats (249).

Oxidative stress is often interrelated with the disrupted Ca^2+^ homeostasis, in mitochondria further affecting OXPHOS and in exaggerated form leading to apoptosis accompanied by the so-called permeability transition opening (29, 95). Oxidative stress leads undoubtedly to impaired maintenance and biogenesis of pancreatic β-cells (see the Impaired Biogenesis of Pancreatic β-Cells section), including the putative “insulin resistance of β-cells,” that is, the dysregulation of autocrine insulin pathway, if it exists. This leads to decreased mtDNA copy number (262) and further GSIS deterioration by such a vicious cycle. Factors of mtDNA replication and transcription are essentially involved and their dysregulation or impairment leads to β-cell dysfunction including GSIS failure (214).

Also, the excessive generation of superoxide and H_2_O_2_ due to the elevated activity of NOXs results in impaired mitochondrial function and OXPHOS, consequently in impaired insulin secretion (207, 212). Elevated ROS also regulate signaling pathways by inhibiting activities of phosphatases and the consequent increase in protein tyrosine phosphorylation levels (22). In turn, redox signaling activates certain kinases, for example, p38 MAPK (234), JNK, PKC (166), and ERK (235). Such signaling often modifies ion channel function. Besides inducing Nrf2 signaling, oxidative stress also interferes with the inflammatory signaling of the NF-κB since the oxidized NF-κB exhibit reduced DNA binding (147). Also, a long-term activation of the 5′ AMP-activated protein kinase (AMPK) decreases GSIS (306).

#### Apoptosis of β-cells resulting from oxidative stress

Phylogenesis developed cell death mechanisms for situations when the above-described stresses would exceed the reparative capacity of tissue or organism. In contrast to the most investigated type of regulated cell death, termed *apoptosis* (118, 183, 199), *necrosis* was characterized as a nonspecific cell death. Recently, *ferroptosis* was classified as another type of cell death, arising from specific signals given by lipid hydroperoxides (266, 282, 307). It has been considered that not the apoptosis, but ferroptosis predominantly causes various neurodegenerative diseases. The role of ferroptosis in β-cells has yet to be investigated. Nevertheless, the disruption of iron homeostasis leads to β-cell death (19, 146).

The two main apoptotic signaling pathways have been described. First, the intrinsic pathway, also known as the mitochondrial pathway, which is activated by different types of cell stress such as hypoxia or oxidative stress, involves proteins of the Bcl-2 family and is manifested by the release of cytochrome *c* and other factors into the cell cytosol (106). The second one is the extrinsic pathway or the pathway mediated by cell death receptors, which involves ligands belonging to the TNF superfamily, such as TNF-α, Fas-L, and its respective receptors expressed on the surface of the pancreatic β-cells that transfer the apoptotic signal downstream through its cell death domain to other associated proteins (80, 242).

Apoptosis has been accepted as a major mechanism addressing β-cell failure in type 2 diabetes (Fig. 4). Oxidative stress and accompanying ER stress seem to be two major players upstream of the apoptotic cascade. Recently, an oxidative stress-responsive apoptosis-inducing protein (ORAIP) was found to induce apoptosis in pancreatic β-cells (304). Oxidative stress can induce β-cell apoptosis by MAPK plus JNK and ERK1/2 phosphorylation (119). The intracellular methylglyoxal accumulating at hyperglycemia was reported to induce the inositol-requiring protein-1 (IRE1)-JNK pathway of apoptosis initiation (184). Glycogen accumulation upon hyperglycemia is also setting conditions for apoptosis (40). In progressed diabetic states, amyloid polypeptide and NOXs are activated by the MAPK-JNK pathway when amyloid polypeptide activates apoptosis *via* the apoptosis signal-regulating kinase-1 (ASK1) (75, 102, 258).

A variety of apoptosis inducers exists. Notably, cytokines, and namely IL-1β, have been shown as important apoptosis mediators (310). Apoptosis as a response to nutrition overload involves the ER stress, but the distal effector mechanisms remain to be uncovered (31). The ER stress was described to be linked to apoptosis in β-cells chronically exposed to elevated FAs (155, 173). The main pathway axis includes the protein phosphatase 1 regulatory subunit 15A also known as growth arrest and DNA damage-inducible protein GADD34 (growth arrest and DNA damage-inducible protein 34); the eukaryotic initiation factor 2-α; the activating transcription factor 4 (ATF4); and protein kinase R (PKR)-like endoplasmic reticulum kinase (PERK; EIF2AK3).

Such signaling through the GADD34 protein-EIF2α-(ATF4)-PERK axis induces oxidative stress. This was reflected by the findings that knockout mice of *db*/*db* background, with the deleted CCAAT-enhancer binding protein homolog (CHOP), are protected against the β-cell oxidative stress. Similarly, other proapoptotic members, such as the p53-upregulated modulator of apoptosis (PUMA), or death protein 5 (DP5)/harakiri (Hrk), were found to be upregulated by palmitate independent of CHOP/ATF4 (64). The FA overload-induced apoptosis was described to be connected to permeability transition pore opening with the ADP/ATP carrier participation. These effects also correlated with the affected Ca^2+^ homeostasis, especially in mitochondria (29, 95). Also, ER stress is involved in parallel with apoptosis as induced by for example, palmitic acid (200).

Apoptosis is prevented by Bcl-2 and Bcl-xL promoting β-cell survival, but Bcl-2 was found also to modulate ROS signaling and suppress a redox-regulated mitochondrial proton leak in β-cells (3). Interestingly, insulin itself *via* elevated H_2_O_2_ promotes β-cell death (250). TXNIP is induced by strong ER stress leading to oxidative stress and inflammation. Interestingly, TXNIP is not induced by chronic lipotoxicity, however, is induced by chronic hyperglycemia (223).

It is well recognized that apoptotic mechanism accompanies cytokine-induced β-cell death mainly during type 1 diabetes but also in obese type 2 diabetic humans (27). However, the rate of β-cell apoptosis is relatively low and thus may not entirely explain the disappearance of β-cell mass *in vivo* during the type 2 diabetes development (142). This indicates that β-cell functional impairment rather than simply β-cell loss largely contributes to the insufficient insulin secretion and glycemic control in type 2 diabetes. Thus, the concept of β-cell de/transdifferentiation as an alternative mechanism of β-cell loss of function seems to be a preferential variant for explanation, as discussed in the β-Cell Biogenesis section.

### Damages to sensing of non-glucose secretagogues

#### FA sensing in pancreatic β-cells—intact *versus* impaired

FAs were considered to only augment GSIS in pancreatic β-cells when exposed at low doses or for hours (49, 92, 99). The existence of FA-stimulated insulin secretion (FASIS) has not been widely recognized; despite it has been traditionally ascribed to the GPR40 pathway (96, 98, 248, 113, 122, 129, 139, 168, 171, 194, 235).

Since this pathway stimulates insulin granule exocytosis *via* the Gq protein or Gs protein plus arrestin routes, it is predominantly K_ATP_-independent. That is why fatty acid-stimulated insulin secretion (FASIS) can exist even without raising glucose above the fasting levels (51, 139). Only the nonreceptor metabolic branch of FASIS involving the glycerol/FA cycle partly contributes to the canonical K_ATP_-dependent pathway of insulin secretion by FA equivalents entering to the mitochondrial FA β-oxidation. Even the glycerol/FA cycle stimulates insulin secretion partly by a receptor pathway, in this case *via* the exocytosis-promoting protein Munc13-1 activated by the released 2/monoacyl glycerol (231).

Ježek *et al.* have identified FA β-oxidation, as one leg in the FASIS mechanism, the branch producing essential H_2_O_2_ from the ETF:QOR-formed superoxide (36, 136); but also revealed a new amplifying mechanism for the GPR40 branch of insulin secretion (providing ∼60% FASIS): amplification by FAs cleaved from mitochondrial phospholipids by the H_2_O_2_-activated iPLA2γ (136), recently confirmed in islets (138, 139, 175) (Fig. 2).

However, the acute lipotoxicity, due to the instantly exaggerated FA levels, is frequently found only *in vitro* (48, 165). It may result from the excessive mitochondrial uncoupling, inhibition of the respiratory chain, or permeability transition pore opening when imbalance with 2-monoacylglycerol metabolism and FA β-oxidation exists (10) (Fig. 4). Sirtuins *via* deacetylation of the forkhead box protein O1 (FoxO1) suppress expression of genes required for FA β-oxidation but do not affect GSIS (162).

Lipotoxicity is typically associated with the oxidative stress. For example, p47phox subunit of NOX was elevated by palmitate (207). The toxicity was exerted by long-chain FAs of total 500 μ*M* concentrations with chain lengths >14, generating H_2_O_2_ reportedly in the peroxisomal β-oxidation (230). In contrast, in human studies, following oral ingestion of the three fat emulsions over 24 hours, plasma FAs were elevated by approximately twofold over the basal level and only ingestion of PUFAs resulted in absolute decrease in GSIS (300). Also, fasting serum FA levels correlated well with the impaired insulin secretion in type 2 diabetes patients (208). Simulations with a FA mixture mimicking *in vivo* situation in MIN6 cells showed decreased viability, reduced antioxidant capacity, and OXPHOS plus elevated lipid peroxidation (285).

Effects of various FAs on β-cell function are complex and pleiotropic, depending on concentration, the chemical nature, including length (62), exposure time, and interaction with other FAs and nutrients (28, 93, 94, 135). For example, high palmitic acid dose at high glucose, that is, under nutritional stress, induced ER stress and profoundly suppressed insulin secretion (57, 115). It can be recognized that most of the FA lipotoxic effects originate from the alternations of cell signaling pathways, often with involved redox signaling and hence accompanied by a parallel oxidative stress. Such altered signaling may subsequently transfer β-cells into more permanent oxidative stress states.

Palmitate impairs expression of the pancreatic and duodenal homeobox 1 (Pdx1) and GLP-1 receptors in β-cells by elevating a sterol regulatory element-binding protein SREBP-1C (210). Chronic palmitate treatment leads to GSIS suppression due to the decreasing activity of *Ins* promoter and binding of Pdx1 and MafA (a member of the basic-leucine-zipper family of transcription factors) to the preproinsulin gene-flanking sequence (108). The insulin exocytosis is also hampered by palmitate-induced dissociation of Ca^2+^ channels from secretory granules (117) and by the impaired cAMP generation (279). Impaired Ca^2+^ homeostasis leads to concerted effects with FAs in oxidative stress induction (188). Promotion of lipotoxicity exists also *via* miRNA, as mediated by PERK/p53-dependent pathway activated by miR-34a-5p upon stearic acid supplementation (187). A novel mechanism of glucolipotoxicity was revealed, which contributes to β-cell dysfunction and cell death. It is initiated by a small GTPase Rac1, which induces β-cell apoptosis by the activation of NOX consequently leading to the increased expression of FA transporter CD36 (81).

#### Sensing of other secretagogues—impaired *versus* intact

For insulin secretion stimulated by keto-acids, redox signaling similar to that existing during FA β-oxidation exists, which is linked to the respiratory chain by ETF:QOR, producing superoxide at the E_F_ site (136). Mitochondrial H_2_O_2_ substantiating the redox signal originates from the MnSOD-converted superoxide. We hypothesize that this redox signaling also activates iPLA2γ and thus cleaves FAs for GPR40 pathway of insulin secretion (Fig. 2).

Leucine, isoleucine, and valine are transported to the mitochondrion where the branched-chain 2-ketoacid amino transferase converts them to 2-keto-isocaproate, 2-keto-3-methyl valerate, and 2-keto-isovalerate, respectively (58, 74). Next, the branched-chain 2-ketoacid dehydrogenase converts them to isovaleryl-CoA, 2-methylbutyryl-CoA, and isobutyryl-CoA, respectively. In the subsequent reaction series resembling β-oxidation, final products are made as acetyl-CoA (acetoacetate), propionyl-CoA (or again acetyl-CoA), and succinyl-CoA, respectively, entering the Krebs cycle and being oxidized by the ETF:QOR. Subsequently, the elevated OXPHOS results in the K_ATP_-dependent insulin secretion.

The mitochondrial dependence of insulin secretion stimulated by keto-acids is reflected by its blockage due to ablation of mitochondrial transcription factor B2 (214). One can predict that interference between insulin secretion stimulated by keto-acids and FASIS exists. Also, the oxidative stress may result from the related metabolism, namely at the FA overload with severe consequences for insulin secretion.

### Systemic oxidative stress affecting β-cells

#### Consequences of chronic high glucose, glucotoxicity

Concentrations out of the physiological glucose stimulatory range, a “GSIS range” (3–11 m*M* in INS-1E cells, approximately 5–10 m*M* in mice and similarly for humans), are harmful especially when exposed to β-cells at prolonged time. Thus, for example, AMPK activation by glucose can contribute to the oxidative stress (251). Physiologically, high glucose inhibits AMPK which increases MafA and activates *Ins* promoter (132 [retracted]). Glucose higher than 10 m*M* stimulates β-cell mass maintenance by stimulating proliferation, neogenesis, and hypertrophy (24), and, as previously interpreted, also *via* autocrine insulin signaling (38, 170, 186, 220).

The term *glucotoxicity* is referred to, when much higher chronic glucose levels, pathological effects that overwhelm the beneficial glucose “maintenance effects.” A hallmark of glucotoxicity is a profound oxidative stress due to inhibition of protein expression for antioxidant defense (124, 153) (Fig. 4). Thus, hyperglycemia activates JNK and impairs the function of Pdx1 and induces ER stress (223).

Another hallmark of glucotoxicity originates from the spontaneous reactions of glucose and other sugars with amine residues of proteins, lipids, and nucleic acids forming the so-called AGE (61, 121). Polyol pathway is activated when excess glucose is converted to sorbitol in the presence of aldose reductase, consuming NADPH and thus contributing to prooxidation state (109). Enhanced glucose influx *via* the so-called hexosamine pathway induces O-glycosylation of signaling molecules that then leads to dysregulation of a crucial survival pathway leading to oxidative stress. The crucial pathway is the insulin receptor and insulin receptor substrates/PI3 kinase pathway (154). Moreover, hyperglycemia increases diacylglycerol, which activates PKC inducing NOX activity and ROS production (127).

Finally, persistent hyperglycemia and accompanying enhanced ROS exposure activate glycosylation, leading to protein, lipids, and nucleic acids unfolding. Specifically, one polypeptide termed amylin is affected this way in islets in type 2 diabetes. The resulting antiparallel crossed β-pleated sheet structure amyloid is then sensitive to free radical polymerization and promotes significant β-cell cytotoxicity (1, 205, 298).

#### Cytotoxicity

The diverse molecular mechanisms involved in the pancreatic β-cell cytotoxicity and death include an above-described apoptotic process, which participates in the immune-mediated insulitis in type 1 diabetes mellitus. Among these cytotoxic mechanism, one can emphasize the action of proinflammatory cytokines, ROS production leading to oxidative stress, DNA fragmentation (typical of necroptosis in type 1 diabetic patients), excessive production of amyloid polypeptide, induction of ER stress, disruption in autophagy mechanisms, and initiation of the inflammasome formation [recently reviewed in Rojas *et al*. (242)].

A systemic source of proinflammatory cytokines may be WAT or immune cells. Proinflammatory cytokines are involved in provoking the pathogenesis of tissue-specific IR by interfering with the insulin signaling pathways (239). Endocrine and paracrine β-cell signaling responses on systemic cytokines are similar and have been frequently mimicked *in vitro*. The proinflammatory cytokines have been shown to cause a concentration and time-dependent effects disrupting pancreatic β-cell function and viability. To reproduce the acute regulatory effects of systemic inflammation on β-cell secretory responses, these cytokines were titrated starting at femtomolar concentrations and incubated for 14–16 hours (27).

**FIG. 5.**
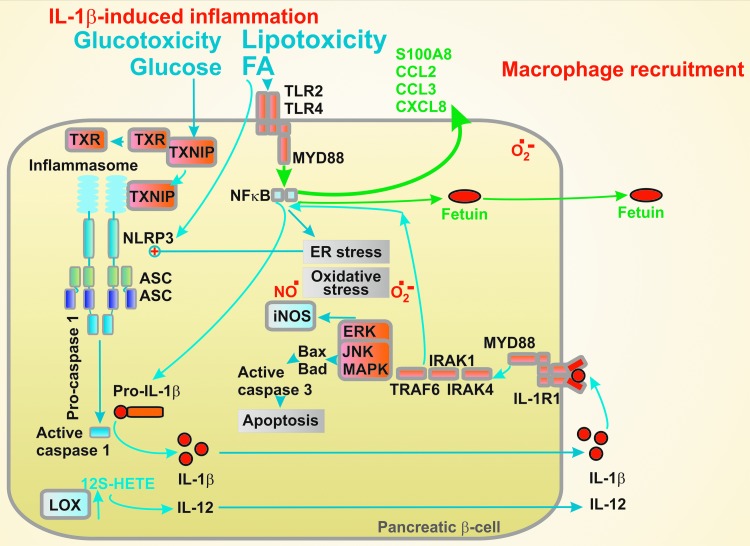
**β-cell can transfer glucotoxicity and lipotoxicity toward inflammation.** Glucose can lead to the TXNIP-initiated NLRP3 inflammasome assembly even in pancreatic β-cells, which is also activated upon the ER stress. This can increase expression and excretion of interleukins, such as IL-1β. Paracrine or autocrine action of IL-1β stimulates interleukin-1 receptor-1 (IL-1R1) signaling further revolving the oxidative stress and proapoptotic state. Lipotoxic FAs *via* TLR2 and TLR4 can stimulate both inflammasome formation and NF-κB signaling, initiating besides other factors, fetuin and chemokines. These provide signals for macrophage recruitment and concomitant islet inflammation. Hypothetically, these signals can be relayed up to periphery to induce insulin resistance. IL, interleukin; IL-1β, interleukin-1β; NF-κB, nuclear factor kappa-light-chain-enhancer of activated B cells; NLRP, NOD-like receptor containing pyrin domain; TLR, toll-like receptor; TXNIP, thioredoxin interacting protein.

In contrast, to illustrate the adverse effects of local chronic islet inflammation, either higher concentrations or longer times (more than 16 hours of exposure) were used (27). It has been concluded that the systemic (or paracrine or autocrine, *i.e*., extracellular for β-cell) inflammatory responses activate the β-cell transcription of various proinflammatory mediators, notably cytokines/chemokines, with involvement of various signaling pathways, oxidative and metabolic stress. The outcome lies also in the recruitment of macrophages to pancreatic islets (Fig. 5).

Consequently, if cytokines excreted by β-cells are low (drained away by the blood flow) that cannot contribute to the β-cell death, the acceleration of islet inflammation by the recruited macrophages increases the probability of the apoptosis. The most prominent apoptotic pathways involved are the NF-κB and MAPK pathways.

Using the human EndoC-βH1 β-cell line, a detailed analysis of cytokine toxicity was performed (105). In this study, the most prominent cytotoxic effect was observed with a mixture of three cytokines (IL-1β, TNF-α, and IFNγ), while any of single cytokine alone had either no or slight effect on the caspase-3 activation. The treatment of cytokines caused activation of NF-κB, *Nrf1*/*2* gene expression, and ROS formation. However, the ER stress markers were not induced. In general, cytokines can induce the ER stress and further induce β-cell apoptosis. However, it is still unclear whether the ER stress is required to elicit the cytokine-induced β-cell apoptosis. In any case, the cytokine treatment caused a significant suppression of the insulin secretory response, as documented by significant reduction of ATP and the absence of ATP/ADP ratio elevations as responding to glucose.

Consequently, GSIS was abolished by the proinflammatory cytokines, but without induction of IL-1β expression. Cytokine-mediated caspase-3 activation was accompanied by the increased ROS formation while cytokines transiently increased the expression of UPR genes, without inducing ER stress-marker genes (105).

NOX1 plays a key role in cytokine-induced β-cell dysfunction (295). Plasmid-driven elevation of NOX1 resulted in elevated ROS, loss of GSIS, and increased apoptosis, with outcomes analogous to cytokine treatment. In contrast, the lowering of NOX1 expression conferred protection to β-cells and islets from the damaging effects of inflammatory cytokines (295).

Serum levels of the alpha-cytoplasmic isoform of heat shock protein 90 (hsp90) were shown to be elevated in individuals with new onset type 1 diabetes (294), and a detailed study showed that human β-cell lines and cadaveric islets released hsp90α in response to stress induced by treatment with a combination of proinflammatory cytokines, including IL-1β, TNF-α, and interferon-γ (218). The hsp90α release was found to be driven by cytokine-induced ER stress mediated by JNK, a pathway that can eventually lead to β-cell apoptosis (218).

The proinflammatory signaling also include TLR4, which has been directly associated with β-cell dysfunction (63). The TLR4 receptor is activated by exposure to lipopolysaccharides (LPS), which are a major component of the outer membrane of gram-negative bacteria. LPS have been shown to trigger the upregulation of inflammatory cytokines, increase ROS production, and decrease insulin secretion in INS-1 pancreatic β-cells (73). The inhibition of TLR4 partially prevented LPS-induced dysfunction of rat islets (290).

## Impaired Biogenesis of Pancreatic β-Cells

### Heterogeneity and plasticity of pancreatic islets

#### Is there only a single β-cells phenotype?

In type 2 diabetes development, a significant decrease in the β-cell mass occurs not only due to the cell death but also due to β-cell dedifferentiation/transdifferentiation. Upon the dedifferentiation, β-cells regress to a less differentiated state or even a precursor-like state, whereas the other hormonal cell type is established during transdifferentiation. Transdifferentiation of β-cells to α-cells occurs in diabetic animals and patients (39, 54, 264, 265).

The dedifferentiation is characterized by downregulation of gene expression of *Mafa*, *Neurod1*, *Nkx6.1*, and *Foxo1* genes encoding β-cell-specifying transcription factors, plus downregulation of insulin expression and expression of genes encoding processing and secretory pathway proteins (*Hspa8*, *Dnaja2*, *Ssr1*, *Sec13*, *Copb2*). In contrast, expression of atypical or “disallowed” genes for β-cells is enhanced by removing their suppression. For example, lactate dehydrogenase, hexokinase-1, monocarboxylate transporter MCT1, or progenitor cell genes (*Neurog3*, *Pax4*, *Sox9*) are induced. These alterations lead to phenotype changes, undoubtedly causing the deterioration or the lack of insulin secretion.

Although the mechanism of initiation is not fully understood, one of the stimuli is the glucotoxicity as such (24). In contrast, normal glucose metabolism is a major regulator of the β-cell differentiated phenotype due to the identity checking mechanism(s) (see the β-cell biogenesis section). Practically, model β-cell cultures have optimum maintenance at about 10 m*M* glucose (201), which increases messenger RNAs (mRNAs) of critical genes for insulin secretion and preceding metabolic pathways, including *Glut2*, *Kir6.2*, and *Sur1* (25).

The question arises, how to really define the β-cell? Is it a cell with the active *INS* promoter, independent of transcribing other genes in different pattern? Alternatively, is there only a single ideal transcriptome pattern that defines the real β-cells having intact and presumably maximum GSIS? Swisa *et al.* defined the β-cell as a cell “capable of synthesizing, processing and secreting mature insulin in response to metabolic, hormonal and neurologic stimuli, or on a molecular level as a cell that expresses the full complement of genes associated with normal, regulated insulin secretion” (270).

Recent findings rather show that a spectrum of β-cells exists not only during ontogenesis but even after finished islet biogenesis. Such “flexible” biogenesis results in the existence of different states of maturation for β-cells. Undoubtedly, further remodeling of β-cells exists during the pathogenesis development. One could classify this as the initiating compensation responses, and after their failure, escape responses leading either to dedifferentiation or to transdifferentiation. We will briefly describe this in the next sections, leaving out the insulin promoter interactions and insulin expression that would deserve a self-standing review.

#### β-cell biogenesis

Neonatal β-cells are functionally incompetitive, whereas in adult β-cells, the replication is hindered but GSIS is fully functional. The transition between these limiting states can be reversed by the physiological non-oncogenic levels of c-Myc (233). Importantly, redox regulations are also involved in β-cell biogenesis.

Indeed, decreasing ROS levels were correlated with the diminished β-cell differentiation. In pancreatic explants cultures, increased ROS stimulated differentiation from progenitors by affecting ERK1/2 signal pathways (116). In turn, manipulations leading to decreases in cellular ROS levels prevented normal β-cell differentiation. Moderate increases in cytosolic H_2_O_2_ were found to promote β-cell proliferation in zebrafish, whereas high H_2_O_2_ levels can stimulate differentiation of new β-cells from progenitors (8). It has been hypothesized that the moderate H_2_O_2_ levels are necessary for β-cell proliferation. This may happen also during overnutrition in compensation by the increased β-cell mass.

Also miRNAs significantly regulate β-cell development, proliferation, insulin biosynthesis, and secretory processes, in addition to dedifferentiation or transdifferentiation (66). In contrast a different miRNA set (miR-7a, miR-132, miR-212) is involved in β-cell dysfunction by hyperglycemia and type 2 diabetes development. Interestingly, some of the so-called β-cell-disallowed genes are targets of miRNA and their expression is thus excluded. Thus, for example, miR-29a/b targets Slc16a1 gene encoding the monocarboxylate transporter MCT1 (70). Also, miR-328 and miR-384 were found specific for β-cells (46).

#### Regulation of insulin expression

The *INS* gene enhancer region is not only the site enhancing *INS* expression but also provides an important identity check for β-cells (see the β-Cell Identity Checking section). Sequence elements A, C1, C2, E1, E2, Z, and the cAMP-response element (CRE) have been identified in the *INS* gene enhancer, containing ∼400 bp-spanning binding sites essential for transcription factors (90).

The *INS* gene encodes a 110-amino acid precursor preproinsulin. Cytosolic ribonucleoprotein signal recognition particles enable translocation across the rough ER into the lumen upon cleavage by the signal peptidase, thus yielding proinsulin. Further ER processing involves the formation of three disulfide bonds to yield insulin (90).

Interestingly, the profound regulation of insulin exists at the translation level. The preproinsulin translation is controlled by glucose (283). Glucose added to primary β-cells thus initially recruits polyribosome complexes with preproinsulin mRNA, which causes up to 30 minutes lag when studied experimentally. However, subsequently after the lag, proinsulin biosynthesis can be increased up to 10-fold. Note that in humans resulting 51 amino acid insulin (5.8 kDa) is associated with the granules with ∼50 other secretory granule proteins. Insulin stored in β-cells is packed in these granules in an insoluble crystalline hexameric form of ∼40 m*M* concentration.

#### β-cell identity checking

**FIG. 6.**
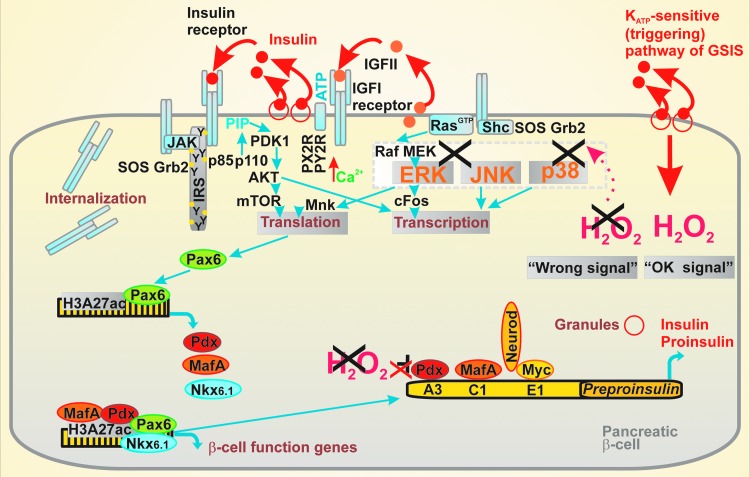
**Identity checks in β-cell involving IRS-1 and K_ATP_ triggering pathway.** Based on the model suggested by Swisa *et al.* (23, 270, 273), the scheme summarizes possible existence of H_2_O_2_-mediated self-checking of the β-cell identity by its proper GSIS function under which cytosolic H_2_O_2_ formation is probably elevated. The elevated H_2_O_2_ substantiates redox signaling, which by yet unknown means provides benefits to β-cell “well-being.” The “wrong” or “incorrect” signal is obtained if the impaired GSIS or other β-cell functions stop such a redox signaling. These benefits may be obtained in parallel by a putative autocrine insulin, autocrine IGF, and paracrine and endocrine GLP-1 (GIP) signaling. Insulin and IGF signaling meets at the IRS signaling promoting transcription and translation of Pax6, a master factor, which further stimulates the expression of the β-cell identity genes (*Pdx, MafA, Nkx6.1*), which consequently maintain proper function of β-cell, especially intact expression of preproinsulin gene and proteins of insulin granules. GIP, gastric inhibitory polypeptide; GLP-1, glucagon-like peptide 1; IGF, insulin-like growth factor; IRS, insulin receptor substrate; Pdx, pancreatic and duodenal homeobox.

The direct transcriptional activation of β-cell-specific genes and repression of β-cell-disallowed genes substantiate the basic mechanism of checking the identity of pancreatic β-cells. Thus, for example, Pdx1 represses MafB preventing (trans)differentiation into α-cells (91). Nkx6.1 represses numerous genes including somatostatin gene (277). Nkx2.2 mRNA levels were found to be increased at 10 m*M* glucose *versus* nonstimulating 2–5 m*M* glucose (25). However, the master regulator of β-cell-specific genes is Pax6 (269) (Fig. 6). Interestingly, transdifferentiation into β-cells also exists from the other islet cell types. Specific epigenetic pattern is most likely locking these cells as endocrine islet cells, which never transdifferentiate beyond their lineage (270).

We may introduce a wider concept of the β-cell identity checking, which by homeostatic coordination of external factors and autocrine factors maintains optimum insulin gene expression, epigenetics, and insulin secretion on nutritional stimuli. When self-checking receives signals of the functional impairment, for example, an insufficient autocrine function, the consequences usually lead to cell death initiation or escape from it by de/transdifferentiation.

Certain modes of the β-cell identity checking also involve redox signaling or redox regulations. For example, Pdx and MafA as the main β-cell differentiation regulators are redox-sensitive (104). Pdx acts in a redox-sensitive manner during ontogenesis and MafA in mature β-cells. Together with the transcription factor NeuroD, Pdx and MafA bind to the *Ins* promoter. Pdx cooperates with the basic helix-loop-helix (bHLH) proteins such as β-2 and E47 that bind to the E1 site of insulin promoter (236).

Thus, both high glucose and ROS induce proinsulin expression as well as GLUT2 expression. The continuous activity of Pdx1-maintained gene expression is essential for mature β-cells (91). Also, Nkx6.1 serves as one line of identity checking, although being formerly considered merely as ontogenic factor important for β-cell differentiation in embryonic stage. Loss of Nkx6.1 leads to transdifferentiation into δ-cells (277). Importantly, hyperglycemia sets conditions when a “wrong” identity signals are received by the identity checking mechanisms (54).

Recently, the canonical pathway of GSIS including K_ATP_, Ca_L_, and being triggered by ATP rise due to OXPHOS elevation has been recognized (and hypothesized) as providing the major redox identity checking signal (270). The ongoing ROS (superoxide and H_2_O_2_) cytosolic release is assumed to substantiate the physiological redox identity checking signal (Fig. 6). Upon its loss (decline of redox signaling), the “wrong” identity signal is imposed (Fig. 6). The molecular nature of this is, however, unknown.

#### Stress-induced dedifferentiation and transdifferentiation of β-cells

A consensus has been established, pointing out that the β-cell functional impairment due to metabolic stress and involving de/transdifferentiation predominantly contributes to type 2 diabetes, rather than outright β-cell loss. Indeed, β-cell de/transdifferentiation occurs, leading to, for example, that 20% of the islet cells found not to express any of five hormonal transcripts and only 34% to be monohormonal (278). The dedifferentiation causes the loss of functional β-cell mass with ultimately defective insulin secretion in type 2 diabetes (273). Notably, dedifferentiation of β-cell is reversible when exposed to normal glucose levels (292). In any case, the β-cell dedifferentiation represents a partial or complete reversible loss of the β-cell identity, such that, for example, gastrin is expressed in certain dedifferentiated cells and can be therefore used as a dedifferentiation marker (54, 65).

However, not only late but also earlier events include cytokine and chemokine secretion by β-cells, recruiting macrophages by stress signaling (77). They reflect the impaired self-maintenance of β-cells, which might be hypothetically considered as the initial event in prediabetes development (Fig. 1). Such β-cell decompensation can also be regulated by miRNAs (66). The β-cell dedifferentiation can also result from the hyperglycemia-induced zinc depletion due to excessive losses of zinc in the secreted insulin granules (172).

When β-cells are dedifferentiated, they become invisible for many common methods of detection. Moreover, cellular differentiation does not need to be unidirectional. In the case of sustained hyperglycemia, β-cells can lose their differentiated phenotype in the process of dedifferentiation, whereas in the late stage of type 2 diabetes, they may even transdifferentiate into α-cells (71) or δ-cells (6). It has been demonstrated for the isolated human pancreatic islets that 20% of the cells do not express any of five hormonal transcripts and only 34% were found to be positive monohormonal (213).

Similarly, the other group demonstrated that 45% of rodent β-cells express multihormonal transcripts (156). Consequently, dedifferentiation in type 2 diabetes causes the effective loss of functional β-cell mass (“true β-cells”) with the ultimately defective insulin secretion. However, there is a period during the earlier development of diabetes, when dedifferentiated β-cells can fix their identity through resumption of expression of β-cell-specific genes if unfavorable conditions of glucose saturation are released.

It is recognized that high glucose levels besides duration of hyperglycemia are the two major upstream regulators of this process. Thus, it has been shown that rat islets and purified β-cells are optimally preserved by ∼10 m*M* glucose, which is above blood glucose levels at fasting, whereas either low (2–5 m*M*) or high (30 m*M*) concentrations cause glucotoxicity that can lead in long term to β-cell dedifferentiation (25, 253).

Also, the monogenic form of type 2 diabetes due to mutations in the K_ATP_ Kir6.2 subunit as well as gene polymorphism resulting in the in-excitable β-cells, thereby affecting insulin secretion, cause the increase of “empty” β-cells lacking the mature β-cell markers and instead expressing multipotency markers reflecting dedifferentiation. Interestingly, attempting to achieve euglycemia, the exogenous insulin supplementation, causes regaining of insulin and re-expression of identity genes in β-cells (292).

The signaling pathway(s) translating conditions of chronically increased glucose to gene transcription leading to de/transdifferentiation also include ER. The strong metabolic load inducing insulin-producing machinery occurring during obesity in compensation for IR might cause severe ER stress and insufficient cellular adaptation to ER stress. Consequently, the concomitant UPR can drastically reduce GSIS.

The ER stress arises from the accumulation of misfolded proteins often caused by the protein overload when the rate of synthesis exceeds the capacity of ER and further protein exit from ER into the secretory pathway. Alternatively, the ER stress results from alterations in the ER milieu that comprises the folding efficiency. We can easily explain why pancreatic β-cells are vulnerable to the ER stress, considering that the proinsulin represents ∼20% of all transcribed mRNA in the β-cell and insulin production occupies around 70% of the protein synthetic machinery.

The failure of adaptive UPR is associated with diabetes progression and affected β-cell differentiation. The adaptive UPR genes (*Hspa5*, *Fkbp11*, *Dnajc3*) are upregulated in prediabetic *db*/*db* mouse islets (age of 6 weeks), but their expression will decrease in diabetic mice (age of 14 weeks). Numerous UPR, ER-Golgi transport, ER quality control, endoplasmic reticulum-associated degradation (ERAD), and retro-translocon-related genes were significantly downregulated in islets of type 2 diabetic donors (45). The defective ER protein folding machinery manifested mainly by major signaling arms of PERK, ATF6, and the serine/threonine–protein kinase/endoribonuclease IRE1 leading to subsequent accumulation of unfolded proteins in ER has been shown to trigger oxidative stress, which induces altered gene expression (18, 110).

Redox status was shown to have an impact on cellular localization of Pax6 in MIA-PaCa2 cells representing the cells from the pancreatic origin. While under low oxidative stress, Pax6 diffused from nucleus to the cytoplasm, under moderate stress, Pax6 moved back to nucleus (257). H_2_O_2_ treatment of β-cell line and isolated islets altered the nuclear expression and DNA binding activity of MafA and Pdx1 (103, 112). Their altered expression was found in the islets of diabetic ZDF rats. The expression was normalized by treatment with GPX mimetic ebselen, thus reflecting the participation of redox signaling (192).

Many redox signaling pathways act through various miR regulations of β-cell identity genes (301). Other genes under redox regulation include *Myc*, *Foxo1*, or an inhibitor of differentiation proteins (26). Under elevated ROS levels, FoxO1 translocates to the nucleus where it enhances *Neurod1* and *Mafa* gene expression (163).

In addition to high glucose, hypoxia and low-grade inflammation were also shown to downregulate UPR genes (52). At the same time, cytokine- and glucotoxicity-induced perturbations in ER calcium luminal content that compromise the overall folding capacity might cause β-cell failure as well (47, 111). Lipotoxicity mainly induced by saturated FAs was shown to disrupt ER-to-Golgi protein trafficking (232). This can lead to ER stress resulting in impaired proinsulin maturation and loss of insulin content. In conclusion, dedifferentiation can serve as the potential adaptive mechanism to escape cell death under chronic hyperglycemia at the price of altered identity and function.

### Impaired β-cell homeostasis and autocrine or paracrine functions

#### Autocrine insulin

Two decades ago, studies with β-cell-specific insulin receptor knockout (βIRKO) mice emphasized the possible role of autocrine insulin on fitness, housekeeping and identity, and correct biogenesis of β-cells (170, 186). In contrast, skeletal muscle-specific insulin receptor knockout mice exhibited IR but normal fasting glucose. This suggested that IR originates from multiorgan deficiency and inflammation cross talk, including that of β-cells (44).

Later, an influential report considered the autocrine insulin function as unlikely and theoretically redundant (241). The previously observed effects such as in βIRKO mice were explained rather by the temporal negative feedback of insulin on stimulated insulin secretion mediated *via* the central nervous system. The arguments against the internalization of insulin receptor have been emphasized if local insulin concentrations were higher (at slow vein drainage). The alternative argument against the autocrine insulin function is a probable very low (picomolar) insulin concentration in the pancreatic artery.

Despite the fact that this controversy has not yet been solved, pancreatic β-cells contain active insulin receptor (14, 15, 38, 170, 186, 220, 271). Consequently, an acute autocrine insulin signaling was suggested to have similar impact as in skeletal muscle and the liver, besides positive effects on β-cell proliferation (38). If exists, the autocrine insulin secretion would be beneficial for the regulation of adult β-cell mass, but the acute effects on insulin secretion itself would be even less probable (37, 120).

βIRKO mice showed increased apoptosis, low proliferative rate, and decreased β-cell mass (170). The insulin receptor was found to be required for islet compensatory growth response to IR (220). Nevertheless, the important role is suggested for downstream insulin receptor substrate IRS-1 (14, 15, 222), emphasizing that another receptor and not the insulin receptor mediates the observed beneficial effects. Consequently, insulin-like growth factor 1 (IGF) may be the true autocrine modulator since its receptor is acting upstream.

There are two parts of insulin signaling through the insulin receptor, the Shc-Ras^GTP^-Raf-1 kinase pathway and the Akt kinase pathway, interlinked by the growth factor receptor-bound protein 2 plus son of sevenless homolog 1 (Grb2-SOS) kinase signaling initiated upon tyrosine phosphorylation of IRS-1 (Fig. 6) (107). Akt is linked to IRS-1 *via* p85 p110 phosphatidyl inositol stimulation of PDK1 and subsequently Akt. Insulin would stimulate primary β-cell proliferation *via* the Shc-Ras^GTP^-Raf-1 kinase pathway and simultaneously suppress apoptosis.

The Akt pathway would enhance β-cell mass and improve glucose tolerance. A signalosome complex of glucokinase, proapoptotic protein, Bcl-2-associated death promoter, BADS, and PKA has been reduced in βIRKO, thus linking the lack of autocrine insulin with the development of type-2 diabetes. The INS-1 cells with stable insulin receptor knockdown after reducing glucose and its subsequent elevation decreased insulin gene expression as well as Pdx1 and GLUT2, suggesting rather a positive role of autocrine insulin (288).

#### Oxidative stress due to altered autocrine function of insulin and other factors

Controversially, studies of mice with mutated insulin receptor have indicated that oxidative stress induced by high-fat diet ceased at impaired insulin receptor signaling, evidencing that such autocrine signaling evokes a certain redox signaling (271). In healthy humans, presumable autocrine insulin has acute (4 hours) effects on GSIS (35). It has been discussed, however, whether central nervous system effects are involved (241).

Also, insulin signaling was found to activate mitochondrial nitric oxide synthase (mtNOS), although the Akt-2/protein-kinase-B mediated phosphorylation in skeletal muscle (86). Released freely permeable nitric oxide, NO^•^, having a half-life of 1–10 seconds, causes a mild oxidative and nitrosative stress accompanied by transiently diminished respiration. NO^•^ can also facilitate glucose to glycogen conversion in the skeletal muscle and liver. Nonpeptidyl mimetic L-783,281-activated insulin receptor through Akt-2/phosphatidylinositol-3-kinase (PI3K) signaling causing inhibition of GSIS and basal insulin secretion in human pancreatic islets (227). Also, recent report on isolated mitochondria showed that insulin signaling regulates mitochondrial function in β-cells (186).

### Stress signals of pancreatic β-cells

We hypothesize that self-checking or identity checking of β-cell is the most essential event regulating the correct housekeeping and phenotype of the “true” β-cell. When such self-checking senses that the less intensive or less frequent autocrine signals occur (37, 120), the absence of beneficial controls by, for example, IRS-1 downstream signaling or GLP-1 (60) probiogenic pathways leads to the release of stress signal. Note that this is the sensing of the absence of self-checking, hence it might be executed as, for example, the lack of previously existing redox signals with consequent termination of downstream redox-activate kinase signaling. Alternatively, the absence of elements promoting *INS* gene expression may substantiate the initiation of putative stress signals (Fig. 6).

Thus, a link may be hypothetically predicted between the deteriorated autocrine signals including that of insulin and low expression of β-cell-specifying factors and hypothetically also between deteriorated endocrine or paracrine incretin action and increased cytokine and chemokine expression in pancreatic β-cells. Since incretins also stimulate ERK, the absence of certain ERK-dependent gene expression of, for example, repressor of the stress gene might substantiate the stress signal. The primary stress signal can be identical to IL-1β-signaling and downstream cytokine and chemokine expression and release from the “unchecked” β-cell. Besides inflammatory signals, exosomes can also carry out the stress signals from pancreatic β-cells to peripheral tissues, such as transduced by miRNAs (150).

Islet inflammation has already been described above. Inflammation is expected to amplify the stress signaling by pancreatic β-cells. Moreover, a cross talk is inevitable between already weak inflammatory states of WAT and skeletal muscle and incorrectly checked β-cells or dedifferentiated β-cells.

## Conclusions

Oxidative stress is traditionally considered as an umbrella for phenomena of multiple changes in β-cell during the type 2 diabetes development. Oxidative stress cannot be separated from numerous other cell pathology events, such as the attempted compensation of β-cell for the increased insulin demand and dynamics of β-cell biogenesis and its “reversal” at dedifferentiation and from changing islet β-cell mass (also due to transdifferentiation) and inflammation.

However, at the initiated pathology, the compensation responses of β-cells despite the delaying pathology progression set a new state when a strong self-checking function might hypothetically send the putative stress signals to the periphery. Subsequent changes together with lasting glucolipotoxicity undoubtedly promote islet inflammatory responses and further spiral of pathology.

Future research should bring an understanding of the β-cell self-checking and putative stress signals since substitution of the “wrong” or “incorrect” signal by the “correct” self-checking signal might help in type 2 diabetes prevention.

Overfeeding, namely the excessive glucose intake, likewise other nutrients acting in a synergy with glucose, has been identified as a major causative origin of numerous events leading to the progressive onset of type 2 diabetes. Both lead to metabolic dysregulations, frequently being related to the oxidative stress in periphery as well as in pancreatic β-cells. All such dysregulations may progress in type 2 diabetes development, being interrelated with the metabolic syndrome.

## Abbreviations Used

βIRKO: β-cell-specific insulin receptor knockout
AGE: advanced glycation end (products)
Akt: protein kinase B
AMPK: 5′ AMP-activated protein kinase
ASC: apoptosis-associated speck-like protein containing CARD
ATF: activating transcription factor
Ca_L_: voltage-gated L-type Ca^2+^channel
CHOP: CCAAT-enhancer binding protein homolog
ER: endoplasmic reticulum
ERK: extracellular-related kinase
ETF:QOR: electron-transferring flavoprotein:Q oxidoreductase
FAs: (free) fatty acids
FASIS: fatty acid-stimulated insulin secretion
FoxO1: forkhead box protein O1
GADD: growth arrest and DNA damage-inducible protein
GIP: gastric inhibitory polypeptide
GLP-1: glucagon-like peptide 1
GLUT: glucose transporter
GPRs: G-protein-coupled receptors
GPX: glutathione peroxidase
GRX: glutaredoxin
GSH: reduced glutathione
GSIS: glucose-stimulated insulin secretion
HETE: hydroxyeicosatetraenoic acid
hsp90: heat shock protein 90
IDH: isocitrate dehydrogenase
IF1: ATP-synthase inhibitory factor 1
IGF: insulin-like growth factor
IL: interleukin
IL-1β: interleukin-1β
iPLA2γ: calcium-independent phospholipase A2, isoform γ
IR: insulin resistance
IRE1: inositol-requiring protein-1
IRS: insulin receptor substrate
JNK: c-Jun N-terminal kinase
K_ATP_: ATP-sensitive K^+^ channel
Keap1: Kelch-like ECH-associated protein 1
LPS: lipopolysaccharides
MAPK: p38 mitogen-activated protein kinase
MCP: monocyte chemotactic protein (chemokine)
MCT: monocarboxylate transporter
MDH: malate dehydrogenase
ME: malic enzyme
MIF: macrophage migration inhibitory factor
MIP: macrophage inflammatory protein (chemokine)
miRNA: microRNA
mRNA: messenger RNA
mtDNA: mitochondrial DNA
MYD88: myeloid differentiation primary response 88 protein
NF-κB: nuclear factor kappa-light-chain-enhancer of activated B cells
NLRP: NOD-like receptor containing pyrin domain
NOX: NADPH oxidase
Nrf2: nuclear factor erythroid 2-related factor
OXPHOS: oxidative phosphorylation
PDH: pyruvate dehydrogenase
PDK: pyruvate dehydrogenase kinase
Pdx: pancreatic and duodenal homeobox
PERK: protein kinase R (PKR)-like endoplasmic reticulum kinase
PKA: protein kinase A
PKC: protein kinase C
PRX: peroxiredoxin
PUFA: polyunsaturated fatty acid
PUMA: p53-upregulated modulator of apoptosis
ROS: reactive oxygen species
S100A8: S100 calcium-binding protein A8
SOD: superoxide dismutase
TLR: toll-like receptor
TNF-α: tumor necrosis factor-α
TRX: thioredoxin
TXNIP: thioredoxin interacting protein
UCP: uncoupling protein
UPR: unfolded protein response
WAT: white adipose tissue

## References

[1] Abedini A, Derk J, and Schmidt AM The receptor for advanced glycation endproducts is a mediator of toxicity by IAPP and other proteotoxic aggregates: establishing and exploiting common ground for novel amyloidosis therapies. Protein Sci 27: 1166–1180, 2018 29664151 10.1002/pro.3425 PMC6032365

[2] Aguirre V, Uchida T, Yenush L, Davis R, and White MF The c-Jun NH(2)-terminal kinase promotes insulin resistance during association with insulin receptor substrate-1 and phosphorylation of Ser307. J Biol Chem 275: 9047–9054, 2000 10722755 10.1074/jbc.275.12.9047

[3] Aharoni-Simon M, Shumiatcher R, Yeung A, Shih AZL, Dolinsky VW, Doucette CA, and Luciani DS Bcl-2 regulates reactive oxygen species signaling and a redox-sensitive mitochondrial proton leak in mouse pancreatic β-cells. Endocrinology 157: 2270–2281, 2016 27070098 10.1210/en.2015-1964

[4] Aizawa T, Yamauchi K, and Yamada M Longitudinal changes in insulin sensitivity, insulin secretion, beta cell function and glucose effectiveness during development of non-diabetic hyperglycemia in a Japanese population. Springerplus 3: 1–6, 2014 24892003 10.1186/2193-1801-3-252 PMC4039663

[5] Akhmedov D, De Marchi U, Wollheim CB, and Wiederkehr A Pyruvate dehydrogenase E1α phosphorylation is induced by glucose but does not control metabolism-secretion coupling in INS-1E clonal β-cells. Biochim Biophys Acta 1823: 1815–1824, 2012 22809973 10.1016/j.bbamcr.2012.07.005

[6] Alán L, Olejár T, Cahová M, Zelenka J, Berková Z, Smětáková M, Saudek F, Matěj R, and Ježek P Delta cell hyperplasia in adult Goto-Kakizaki (GK/MolTac) diabetic rats. J Diabetes Res 2015: 385395, 2015 26236746 10.1155/2015/385395 PMC4506919

[7] Alejandro EU, Gregg B, Blandino-Rosano M, Cras-Méneur C, and Bernal-Mizrachi E Natural history of β-cell adaptation and failure in type 2 diabetes. Mol Aspects Med 42: 19–41, 2015 25542976 10.1016/j.mam.2014.12.002 PMC4404183

[8] Alfar EA, Kirova D, Konantz J, Birke S, Mansfeld J, and Ninov N Distinct levels of reactive oxygen species coordinate metabolic activity with beta-cell mass plasticity. Sci Rep 7: 3994, 2017 28652605 10.1038/s41598-017-03873-9 PMC5484671

[9] Anderson KA, Huynh FK, Fisher-Wellman K, Stuart JD, Peterson BS, Douros JD, Wagner GR, Thompson JW, Madsen AS, Green MF, Sivley RM, Ilkayeva OR, Stevens RD, Backos DS, Capra JA, Olsen CA, Campbell JE, Muoio DM, Grimsrud PA, and Hirschey MD SIRT4 is a lysine deacylase that controls leucine metabolism and insulin secretion. Cell Metab 25: 838.e15–855.e15, 2017 28380376 10.1016/j.cmet.2017.03.003 PMC5444661

[10] Ansari IH, Longacre MJ, Stoker SW, Kendrick MA, O'Neill LM, Zitur LJ, Fernandez LA, Ntambi JM, and MacDonald MJ Characterization of Acyl-CoA synthetase isoforms in pancreatic beta cells: gene silencing shows participation of ACSL3 and ACSL4 in insulin secretion. Arch Biochem Biophys 618: 32–43, 2017 28193492 10.1016/j.abb.2017.02.001 PMC5445904

[11] Archer SL, Wu XC, Thébaud B, Moudgil R, Hashimoto K, and Michelakis ED O_2_ sensing in the human ductus arteriosus: redox-sensitive K^+^ channels are regulated by mitochondria-derived hydrogen peroxide. Biol Chem 385: 205–216, 2004 15134333 10.1515/BC.2004.014

[12] Arredouani A, Ruas M, Collins SC, Parkesh R, Clough F, Pillinger T, Coltart G, Rietdorf K, Royle A, Johnson P, Braun M, Zhang Q, Sones W, Shimomura K, Morgan AJ, Lewis AM, Chuang KT, Tunn R, Gadea J, Teboul L, Heister PM, Tynan PW, Bellomo EA, Rutter GA, Rorsman P, Churchill GC, Parrington J, and Galione A Nicotinic acid adenine dinucleotide phosphate (NAADP) and endolysosomal two-pore channels modulate membrane excitability and stimulus-secretion coupling in mouse pancreatic β cells. J Biol Chem 290: 21376–21392, 2015 26152717 10.1074/jbc.M115.671248 PMC4571866

[13] Ashcroft FMM and Rorsman P Diabetes mellitus and the β cell: the last ten years. Cell 148: 1160–1171, 2012 22424227 10.1016/j.cell.2012.02.010 PMC5890906

[14] Aspinwall CA, Qian WJ, Roper MG, Kulkarni RN, Kahn CR, and Kennedy RT Roles of insulin receptor substrate-1, phosphatidylinositol 3-kinase, and release of intracellular Ca^2+^ stores in insulin-stimulated insulin secretion in β-cells. J Biol Chem 275: 22331–22338, 2000 10764813 10.1074/jbc.M909647199

[15] Assmann A, Ueki K, Winnay JN, Kadowaki T, and Kulkarni RN Glucose effects on beta-cell growth and survival require activation of insulin receptors and insulin receptor substrate 2. Mol Cell Biol 29: 3219–3228, 2009 19273608 10.1128/MCB.01489-08 PMC2682019

[16] Azevedo-Martins AK, Lortz S, Lenzen S, Curi R, Eizirik DL, and Tiedge M Improvement of the mitochondrial antioxidant defense status prevents cytokine-induced nuclear factor-kappaB activation in insulin-producing cells. Diabetes 52: 93–101, 2003 12502498 10.2337/diabetes.52.1.93

[17] Bachnoff N, Trus M, and Atlas D Alleviation of oxidative stress by potent and selective thioredoxin-mimetic peptides. Free Radic Biol Med 50: 1355–1367, 2011 21377525 10.1016/j.freeradbiomed.2011.02.026

[18] Back SH, Scheuner D, Han J, Song B, Ribick M, Wang J, Gildersleeve RD, Pennathur S, and Kaufman RJ Translation attenuation through eIF2α phosphorylation prevents oxidative stress and maintains the differentiated state in β cells. Cell Metab 10: 13–26, 2009 19583950 10.1016/j.cmet.2009.06.002 PMC2742645

[19] Backe MB, Moen IW, Ellervik C, Hansen JB, and Mandrup-Poulsen T Iron regulation of pancreatic beta-cell functions and oxidative stress. Annu Rev Nutr 36: 241–273, 2016 27146016 10.1146/annurev-nutr-071715-050939

[20] Barazzoni R, Gortan Cappellari G, Ragni M, and Nisoli E Insulin resistance in obesity: an overview of fundamental alterations. Eat Weight Disord 23: 149–157, 2018 29397563 10.1007/s40519-018-0481-6

[21] Baron AD, Brechtel G, Wallace P, and Edelman SV Rates and tissue sites of non-insulin- and insulin-mediated glucose uptake in humans. Am J Physiol 255: E769–E774, 1988 3059816 10.1152/ajpendo.1988.255.6.E769

[22] Bedard K and Krause KH The NOX family of ROS-generating NADPH oxidases: physiology and pathophysiology. Physiol Rev 87: 245–313, 2007 17237347 10.1152/physrev.00044.2005

[23] Bensellam M, Jonas JC, and Laybutt DR Mechanisms of β-cell dedifferentiation in diabetes: recent findings and future research directions. J Endocrinol 236: R109–R143, 2018 29203573 10.1530/JOE-17-0516

[24] Bensellam M, Laybutt DR, and Jonas JC The molecular mechanisms of pancreatic beta-cell glucotoxicity: recent findings and future research directions. Mol Cell Endocrinol 364: 1–27, 2012 22885162 10.1016/j.mce.2012.08.003

[25] Bensellam M, Van Lommel L, Overbergh L, Schuit FC, and Jonas JC Cluster analysis of rat pancreatic islet gene mRNA levels after culture in low-, intermediate- and high-glucose concentrations. Diabetologia 52: 463–476, 2009 19165461 10.1007/s00125-008-1245-z

[26] Bensellam M, Montgomery MK, Luzuriaga J, Chan JY, and Laybutt DR Inhibitor of differentiation proteins protect against oxidative stress by regulating the antioxidant–mitochondrial response in mouse beta cells. Diabetologia 58: 758–770, 2015 25636209 10.1007/s00125-015-3503-1

[27] Berchtold LA, Prause M, Størling J, and Mandrup-Poulsen T Cytokines and pancreatic β-cell apoptosis. Adv Clin Chem 75: 99–158, 2016 27346618 10.1016/bs.acc.2016.02.001

[28] Bermudez B, Ortega-Gomez A, Varela LM, Villar J, Abia R, Muriana FJG, and Lopez S Clustering effects on postprandial insulin secretion and sensitivity in response to meals with different fatty acid compositions. Food Funct 5: 1374–1380, 2014 24752559 10.1039/c4fo00067f

[29] Bernardi P, Rasola A, Forte M, and Lippe G The mitochondrial permeability transition pore: channel formation by F-ATP synthase, integration in signal transduction, and role in pathophysiology. Physiol Rev 95: 1111–1155, 2015 26269524 10.1152/physrev.00001.2015 PMC4600949

[30] Bessesen DH The role of carbohydrates in insulin resistance. J Nutr 131: 2782S–2786S, 2001 11584106 10.1093/jn/131.10.2782S

[31] Biden TJ, Boslem E, Chu KY, and Sue N Lipotoxic endoplasmic reticulum stress, β cell failure, and type 2 diabetes mellitus. Trends Endocrinol Metab 25: 389–398, 2014 24656915 10.1016/j.tem.2014.02.003

[32] Bindokas VP, Kuznetsov A, Sreenan S, Polonsky KS, Roe MW, and Philipson LH Visualizing superoxide production in normal and diabetic rat islets of Langerhans. J Biol Chem 278: 9796–9801, 2003 12514170 10.1074/jbc.M206913200

[33] Böni-Schnetzler M, Boller S, Debray S, Bouzakri K, Meier DT, Prazak R, Kerr-Conte J, Pattou F, Ehses JA, Schuit FC, and Donath MY Free fatty acids induce a proinflammatory response in islets via the abundantly expressed interleukin-1 receptor I. Endocrinology 150: 5218–5229, 2009 19819943 10.1210/en.2009-0543

[34] Böni-Schnetzler M, Thorne J, Parnaud G, Marselli L, Ehses JA, Kerr-Conte J, Pattou F, Halban PA, Weir GC, and Donath MY Increased interleukin (IL)-1β messenger ribonucleic acid expression in β-cells of individuals with type 2 diabetes and regulation of IL-1β in human islets by glucose and autostimulation. J Clin Endocrinol Metab 93: 4065–4074, 2008 18664535 10.1210/jc.2008-0396 PMC2579638

[35] Bouche C, Lopez X, Fleischman A, Cypess AM, O'Shea S, Stefanovski D, Bergman RN, Rogatsky E, Stein DT, Kahn CR, Kulkarni RN, and Goldfine AB Insulin enhances glucose-stimulated insulin secretion in healthy humans. Proc Natl Acad Sci U S A 107: 4770–4775, 2010 20176932 10.1073/pnas.1000002107 PMC2842068

[36] Brand MD Mitochondrial generation of superoxide and hydrogen peroxide as the source of mitochondrial redox signaling. Free Radic Biol Med 100: 14–31, 2016 27085844 10.1016/j.freeradbiomed.2016.04.001

[37] Braun M, Ramracheya R, and Rorsman P Autocrine regulation of insulin secretion. Diabetes Obes Metab 14: 143–151, 2012 22928575 10.1111/j.1463-1326.2012.01642.x

[38] Brennand K, Huangfu D, and Melton D All β cells contribute equally to islet growth and maintenance. PLoS Biol 5: e163, 2007 17535113 10.1371/journal.pbio.0050163 PMC1877817

[39] Brereton MF, Iberl M, Shimomura K, Zhang Q, Adriaenssens AE, Proks P, Spiliotis II, Dace W, Mattis KK, Ramracheya R, Gribble FM, Reimann F, Clark A, Rorsman P, and Ashcroft FM Reversible changes in pancreatic islet structure and function produced by elevated blood glucose. Nat Commun 5: 4639, 2014 25145789 10.1038/ncomms5639 PMC4143961

[40] Brereton MF, Rohm M, Shimomura K, Holland C, Tornovsky-Babeay S, Dadon D, Iberl M, Chibalina MV, Lee S, Glaser B, Dor Y, Rorsman P, Clark A, and Ashcroft FM Hyperglycaemia induces metabolic dysfunction and glycogen accumulation in pancreatic β-cells. Nat Commun 7: 13496, 2016 27882918 10.1038/ncomms13496 PMC5123088

[41] Broche B, Fradj S Ben, Aguilar E, Sancerni T, Bénard M, Makaci F, Berthault C, Scharfmann R, Alves-Guerra MC, and Duvillié B Mitochondrial protein UCP2 controls pancreas development. Diabetes 67: 78–84, 2018 29079704 10.2337/db17-0118

[42] Brozzi F, Nardelli TR, Lopes M, Millard I, Barthson J, Igoillo-Esteve M, Grieco FA, Villate O, Oliveira JM, Casimir M, Bugliani M, Engin F, Hotamisligil GS, Marchetti P, and Eizirik DL Cytokines induce endoplasmic reticulum stress in human, rat and mouse beta cells via different mechanisms. Diabetologia 58: 2307–2316, 2015 26099855 10.1007/s00125-015-3669-6

[43] Brüning JC, Gautam D, Burks DJ, Gillette J, Schubert M, Orban PC, Klein R, Krone W, Müller-Wieland D, and Kahn CR Role of brain insulin receptor in control of body weight and reproduction. Science 289: 2122–2125, 2000 11000114 10.1126/science.289.5487.2122

[44] Brüning JC, Michael MD, Winnay JN, Hayashi T, Hörsch D, Accili D, Goodyear LJ, and Kahn CR A muscle-specific insulin receptor knockout exhibits features of the metabolic syndrome of NIDDM without altering glucose tolerance. Mol Cell 2: 559–569, 1998 9844629 10.1016/s1097-2765(00)80155-0

[45] Bugliani M, Liechti R, Cheon H, Suleiman M, Marselli L, Kirkpatrick C, Filipponi F, Boggi U, Xenarios I, Syed F, Ladriere L, Wollheim C, Lee MS, and Marchetti P Microarray analysis of isolated human islet transcriptome in type 2 diabetes and the role of the ubiquitin-proteasome system in pancreatic beta cell dysfunction. Mol Cell Endocrinol 367: 1–10, 2013 23246353 10.1016/j.mce.2012.12.001

[46] van de Bunt M, Gaulton KJ, Parts L, Moran I, Johnson PR, Lindgren CM, Ferrer J, Gloyn AL, and McCarthy MI The miRNA profile of human pancreatic islets and beta-cells and relationship to type 2 diabetes pathogenesis. PLoS One 8: e55272, 2013 23372846 10.1371/journal.pone.0055272 PMC3555946

[47] Cardozo AK, Ortis F, Storling J, Feng YM, Rasschaert J, Tonnesen M, Van Eylen F, Mandrup-Poulsen T, Herchuelz A, and Eizirik DL Cytokines downregulate the sarcoendoplasmic reticulum pump Ca^2+^ ATPase 2b and deplete endoplasmic reticulum Ca^2+^, leading to induction of endoplasmic reticulum stress in pancreatic β-cells. Diabetes 54: 452–461, 2005 15677503 10.2337/diabetes.54.2.452

[48] Carlsson C, Borg LAH, and Welsh N Sodium palmitate induces partial mitochondrial uncoupling and reactive oxygen species in rat pancreatic islets in vitro. Endocrinology 140: 3422–3428, 1999 10433196 10.1210/endo.140.8.6908

[49] Carpinelli AR, Picinato MC, Stevanato E, Oliveira HR, and Curi R Insulin secretion induced by palmitate–a process fully dependent on glucose concentration. Diabetes Metab 28: 3S37–3S44; discussion 3S108–3S112, 2002 12688632

[50] Caton PW, Richardson SJ, Kieswich J, Bugliani M, Holland ML, Marchetti P, Morgan NG, Yaqoob MM, Holness MJ, and Sugden MC Sirtuin 3 regulates mouse pancreatic beta cell function and is suppressed in pancreatic islets isolated from human type 2 diabetic patients. Diabetologia 56: 1068–1077, 2013 23397292 10.1007/s00125-013-2851-y

[51] Cen J, Sargsyan E, and Bergsten P Fatty acids stimulate insulin secretion from human pancreatic islets at fasting glucose concentrations via mitochondria-dependent and -independent mechanisms. Nutr Metab (Lond) 13: 59, 2016 27582778 10.1186/s12986-016-0119-5 PMC5006523

[52] Chan JY, Cooney GJ, Biden TJ, and Laybutt DR Differential regulation of adaptive and apoptotic unfolded protein response signalling by cytokine-induced nitric oxide production in mouse pancreatic beta cells. Diabetologia 54: 1766–1776, 2011 21472432 10.1007/s00125-011-2139-z

[53] Chen Y, Morrow JD, and Roberts LJ, 2nd. Formation of reactive cyclopentenone compounds in vivo as products of the isoprostane pathway. J Biol Chem 274: 10863–10868, 1999 10196163 10.1074/jbc.274.16.10863

[54] Cinti F, Bouchi R, Kim-Muller JY, Ohmura Y, Sandoval PR, Masini M, Marselli L, Suleiman M, Ratner LE, Marchetti P, and Accili D Evidence of β-cell dedifferentiation in human type 2 diabetes. J Clin Endocrinol Metab 101: 1044–1054, 2016 26713822 10.1210/jc.2015-2860 PMC4803182

[55] Ciregia F, Bugliani M, Ronci M, Giusti L, Boldrini C, Mazzoni MR, Mossuto S, Grano F, Cnop M, Marselli L, Giannaccini G, Urbani A, Lucacchini A, and Marchetti P Palmitate-induced lipotoxicity alters acetylation of multiple proteins in clonal β cells and human pancreatic islets. Sci Rep 7: 13445, 2017 29044173 10.1038/s41598-017-13908-w PMC5647430

[56] Cnop M, Abdulkarim B, Bottu G, Cunha DA, Igoillo-Esteve M, Masini M, Turatsinze JV, Griebel T, Villate O, Santin I, Bugliani M, Ladriere L, Marselli L, McCarthy MI, Marchetti P, Sammeth M, and Eizirik DL RNA sequencing identifies dysregulation of the human pancreatic islet transcriptome by the saturated fatty acid palmitate. Diabetes 63: 1978–1993, 2014 24379348 10.2337/db13-1383

[57] Cohen G, Shamni O, Avrahami Y, Cohen O, Broner EC, Filippov-Levy N, Chatgilialoglu C, Ferreri C, Kaiser N, and Sasson S Beta cell response to nutrient overload involves phospholipid remodelling and lipid peroxidation. Diabetologia 58: 1333–1343, 2015 25810039 10.1007/s00125-015-3566-z

[58] Cole JT. Metabolism of BBAAs. In: Branched Chain Amino Acids in Clinical Nutrition, edited by Rajendram R, Preedy VR, Patel V. New York, NY: Springer Science, 2015, pp. 13–24

[59] Collier JJ, Sparer TE, Karlstad MD, and Burke SJ Pancreatic islet inflammation: an emerging role for chemokines. J Mol Endocrinol 59: R33–R46, 2017 28420714 10.1530/JME-17-0042 PMC5505180

[60] Cornu M, Modi H, Kawamori D, Kulkarni RN, Joffraud M, and Thorens B Glucagon-like peptide-1 increases β-cell glucose competence and proliferation by translational induction of insulin-like growth factor-1 receptor expression. J Biol Chem 285: 10538–10545, 2010 20145256 10.1074/jbc.M109.091116 PMC2856261

[61] Coughlan MT, Yap FYT, Tong DCK, Andrikopoulos S, Gasser A, Thallas-Bonke V, Webster DE, Miyazaki J, Kay TW, Slattery RM, Kaye DM, Drew BG, Kingwell BA, Fourlanos S, Groop PH, Harrison LC, Knip M, and Forbes JM Advanced glycation end products are direct modulators of β-cell function. Diabetes 60: 2523–2532, 2011 21911745 10.2337/db10-1033 PMC3178291

[62] Cruciani-Guglielmacci C, Bellini L, Denom J, Oshima M, Fernandez N, Normandie-Levi P, Berney XP, Kassis N, Rouch C, Dairou J, Gorman T, Smith DM, Marley A, Liechti R, Kuznetsov D, Wigger L, Burdet F, Lefèvre AL, Wehrle I, Uphues I, Hildebrandt T, Rust W, Bernard C, Ktorza A, Rutter GA, Scharfmann R, Xenarios I, Le Stunff H, Thorens B, Magnan C, and Ibberson M Molecular phenotyping of multiple mouse strains under metabolic challenge uncovers a role for Elovl2 in glucose-induced insulin secretion. Mol Metab 6: 340–351, 2017 28377873 10.1016/j.molmet.2017.01.009 PMC5369210

[63] Cucak H, Mayer C, Tonnesen M, Thomsen LH, Grunnet LG, and Rosendahl A Macrophage contact dependent and independent TLR4 mechanisms induce β-cell dysfunction and apoptosis in a mouse model of type 2 diabetes. PLoS One 9: e90685, 2014 24594974 10.1371/journal.pone.0090685 PMC3940939

[64] Cunha DA, Igoillo-Esteve M, Gurzov EN, Germano CM, Naamane N, Marhfour I, Fukaya M, Vanderwinden JM, Gysemans C, Mathieu C, Marselli L, Marchetti P, Harding HP, Ron D, Eizirik DL, and Cnop M Death protein 5 and p53-upregulated modulator of apoptosis mediate the endoplasmic reticulum stress-mitochondrial dialog triggering lipotoxic rodent and human β-cell apoptosis. Diabetes 61: 2763–2775, 2012 22773666 10.2337/db12-0123 PMC3478544

[65] Dahan T, Ziv O, Horwitz E, Zemmour H, Lavi J, Swisa A, Leibowitz G, Ashcroft FM, In't Veld PIt, Glaser B, and Dor Y Pancreatic β-cells express the fetal islet hormone gastrin in rodent and human diabetes. Diabetes 66: 426–436, 2017 27864307 10.2337/db16-0641 PMC5248995

[66] Dalgaard LT and Eliasson L An “alpha-beta” of pancreatic islet microribonucleotides. Int J Biochem Cell Biol 88: 208–219, 2017 28122254 10.1016/j.biocel.2017.01.009

[67] Dikalova AE, Itani HA, Nazarewicz RR, McMaster WG, Flynn CR, Uzhachenko R, Fessel JP, Gamboa JL, Harrison DG, and Dikalov SI Sirt3 impairment and SOD2 hyperacetylation in vascular oxidative stress and hypertension. Circ Res 121: 564–574, 2017 28684630 10.1161/CIRCRESAHA.117.310933 PMC5562527

[68] Dinič S, Grdovič N, Uskokovič A, Đordevič M, Mihailovič M, Jovanovič JA, Poznanovič G, and Vidakovič M CXCL12 protects pancreatic β-cells from oxidative stress by a Nrf2-induced increase in catalase expression and activity. Proc Jpn Acad Ser B Phys Biol Sci 92: 436–454, 2016 10.2183/pjab.92.436 PMC5328787 27840391

[69] Dlasková A, Engstová H, Špaček T, Kahancová A, Pavluch V, Smolková K, Špačková J, Bartoš M, Hlavatá LP, and Ježek P 3D super-resolution microscopy reflects mitochondrial cristae alternations and mtDNA nucleoid size and distribution. Biochim Biophys Acta 1859: 829–844, 2018 10.1016/j.bbabio.2018.04.013 29727614

[70] Dooley J, Garcia-Perez JE, Sreenivasan J, Schlenner SM, Vangoitsenhoven R, Papadopoulou AS, Tian L, Schonefeldt S, Serneels L, Deroose C, Staats KA, Van Der Schueren B, De Strooper B, McGuinness OP, Mathieu C, and Liston A The microRNA-29 family dictates the balance between homeostatic and pathological glucose handling in diabetes and obesity. Diabetes 65: 53–61, 2016 26696639 10.2337/db15-0770 PMC4876765

[71] Dor Y and Glaser B Beta-cell dedifferentiation and type 2 diabetes. N Engl J Med 368: 572–573, 2013 23388011 10.1056/NEJMcibr1214034

[72] Drews G, Krippeit-Drews P, and Düfer M Electrophysiology of islet cells. Adv Exp Med Biol 654: 115–163, 2010 20217497 10.1007/978-90-481-3271-3_7

[73] Du SC, Ge QM, Lin N, Dong Y, and Su Q ROS-mediated lipopolysaccharide-induced apoptosis in INS-1 cells by modulation of Bcl-2 and Bax. Cell Mol Biol (Noisy-le-grand) 58 (Suppl): OL1654–OL1659, 2012 22455982

[74] Duan Y, Li F, Li Y, Tang Y, Kong X, Feng Z, Anthony TG, Watford M, Hou Y, Wu G, and Yin Y The role of leucine and its metabolites in protein and energy metabolism. Amino Acids 48: 41–51, 2016 26255285 10.1007/s00726-015-2067-1

[75] Dubey R, Minj P, Malik N, Sardesai DM, Kulkarni SH, Acharya JD, Bhavesh NS, Sharma S, and Kumar A Recombinant human islet amyloid polypeptide forms shorter fibrils and mediates β-cell apoptosis via generation of oxidative stress. Biochem J 474: 3915–3934, 2017 29046394 10.1042/BCJ20170323

[76] Edalat A, Schulte-Mecklenbeck P, Bauer C, Undank S, Krippeit-Drews P, Drews G, and Düfer M Mitochondrial succinate dehydrogenase is involved in stimulus-secretion coupling and endogenous ROS formation in murine beta cells. Diabetologia 58: 1532–1541, 2015 25874444 10.1007/s00125-015-3577-9

[77] Eguchi K and Manabe I Macrophages and islet inflammation in type 2 diabetes. Diabetes Obes Metab 15: 152–158, 2013 24003932 10.1111/dom.12168

[78] Eguchi K, Manabe I, Oishi-Tanaka Y, Ohsugi M, Kono N, Ogata F, Yagi N, Ohto U, Kimoto M, Miyake K, Tobe K, Arai H, Kadowaki T, and Nagai R Saturated fatty acid and TLR signaling link β cell dysfunction and islet inflammation. Cell Metab 15: 518–533, 2012 22465073 10.1016/j.cmet.2012.01.023

[79] Eguchi K and Nagai R Islet inflammation in type 2 diabetes and physiology. J Clin Invest 127: 14–23, 2017 28045399 10.1172/JCI88877 PMC5199688

[80] Eisenberg-Lerner A, Bialik S, Simon HU, and Kimchi A Life and death partners: apoptosis, autophagy and the cross-talk between them. Cell Death Differ 16: 966–975, 2009 19325568 10.1038/cdd.2009.33

[81] Elumalai S, Karunakaran U, Lee IK, Moon JS, and Won KC Rac1-NADPH oxidase signaling promotes CD36 activation under glucotoxic conditions in pancreatic beta cells. Redox Biol 11: 126–134, 2017 27912197 10.1016/j.redox.2016.11.009 PMC5133656

[82] Fava GE, Dong EW, and Wu H Intra-islet glucagon-like peptide 1. J Diabetes Complications 30: 1651–1658, 2016 27267264 10.1016/j.jdiacomp.2016.05.016 PMC5050074

[83] Fernández-Díaz CM, Escobar-Curbelo L, López-Acosta JF, Lobatón CD, Moreno A, Sanz-Ortega J, Perdomo G, and Cózar-Castellano I Insulin degrading enzyme is up-regulated in pancreatic βcells by insulin treatment. Histol Histopathol 4: 11997, 2018 10.14670/HH-11-997 29726577

[84] Fernández-Millán E, Martín MA, Goya L, Lizárraga-Mollinedo E, Escrivá F, Ramos S, and Álvarez C Glucagon-like peptide-1 improves beta-cell antioxidant capacity via extracellular regulated kinases pathway and Nrf2 translocation. Free Radic Biol Med 95: 16–26, 2016 26968794 10.1016/j.freeradbiomed.2016.03.002

[85] Fex M, Nicholas LM, Vishnu N, Medina A, Sharoyko VV, Nicholls DG, Spégel P, and Mulder H The pathogenetic role of β-cell mitochondria in type 2 diabetes. J Endocrinol 236: R145–R159, 2018 29431147 10.1530/JOE-17-0367

[86] Finocchietto P, Barreyro F, Holod S, Peralta J, Franco MC, Méndez C, Converso DP, Estévez A, Carreras MC, and Poderoso JJ Control of muscle mitochondria by insulin entails activation of Akt2-mtNOS pathway: imlpications for the metabolic syndrome. PLoS One 3: e1749, 2008 18335029 10.1371/journal.pone.0001749 PMC2258147

[87] Finol-Urdaneta RK, Remedi MS, Raasch W, Becker S, Clark RB, Strüver N, Pavlov E, Nichols CG, French RJ, and Terlau H Block of K v 1.7 potassium currents increases glucose-stimulated insulin secretion. EMBO Mol Med 4: 424–434, 2012 22438204 10.1002/emmm.201200218 PMC3403299

[88] Fridlyand LE and Philipson LH Does the glucose-dependent insulin secretion mechanism itself cause oxidative stress in pancreatic β-cells? Diabetes 53: 1942–1948, 2004 15277370 10.2337/diabetes.53.8.1942

[89] Fu J, Zheng H, Wang H, Yang B, Zhao R, Lu C, Liu Z, Hou Y, Xu Y, Zhang Q, Qu W, and Pi J Protective role of nuclear factor E2-related factor 2 against acute oxidative stress-induced pancreatic β-cell damage. Oxid Med Cell Longev 2015: 639191, 2015 25949772 10.1155/2015/639191 PMC4407529

[90] Fu Z, Gilbert ER, and Liu D Regulation of insulin synthesis and secretion and pancreatic beta-cell dysfunction in diabetes. Curr Diabetes Rev 9: 25–53, 2013 22974359 PMC3934755

[91] Gao T, McKenna B, Li C, Reichert M, Nguyen J, Singh T, Yang C, Pannikar A, Doliba N, Zhang T, Stoffers DA, Edlund H, Matschinsky F, Stein R, and Stanger BZ Pdx1 maintains β cell identity and function by repressing an α cell program. Cell Metab 19: 259–271, 2014 24506867 10.1016/j.cmet.2013.12.002 PMC3950964

[92] Gehrmann W, Elsner M, and Lenzen S Role of metabolically generated reactive oxygen species for lipotoxicity in pancreatic β-cells. Diabetes Obes Metab 12: 149–158, 2010 21029312 10.1111/j.1463-1326.2010.01265.x

[93] Gehrmann W, Würdemann W, Plötz T, Jörns A, Lenzen S, and Elsner M Antagonism between saturated and unsaturated fatty acids in ROS mediated lipotoxicity in rat insulin-producing cells. Cell Physiol Biochem 36: 852–865, 2015 26044490 10.1159/000430261

[94] Giacca A, Xiao C, Oprescu AI, Carpentier AC, and Lewis GF Lipid-induced pancreatic β-cell dysfunction: focus on in vivo studies. Am J Physiol Endocrinol Metab 300: E255–E262, 2011 21119027 10.1152/ajpendo.00416.2010

[95] Giorgio V, Guo L, Bassot C, Petronilli V, and Bernardi P Calcium and regulation of the mitochondrial permeability transition. Cell Calcium 70: 56–63, 2018 28522037 10.1016/j.ceca.2017.05.004

[96] Gorski JN, Pachanski MJ, Mane J, Plummer CW, Souza S, Thomas-Fowlkes BS, Ogawa AM, Weinglass AB, Di Salvo J, Cheewatrakoolpong B, Howard AD, Colletti SL, and Trujillo ME GPR40 reduces food intake and body weight through GLP-1. Am J Physiol Endocrinol Metab 313: E37–E47, 2017 28292762 10.1152/ajpendo.00435.2016

[97] Graaf CD, Donnelly D, Wootten D, Lau J, Sexton PM, Miller LJ, Ahn JM, Liao J, Fletcher MM, Yang D, Brown AJH, Zhou C, Deng J, and Wang MW Glucagon-like peptide-1 and its class B G protein-coupled receptors: a long march to therapeutic successes. Pharmacol Rev 68: 954–1013, 2016 27630114 10.1124/pr.115.011395 PMC5050443

[98] Graciano MF, Valle MM, Curi R, and Carpinelli AR Evidence for the involvement of GPR40 and NADPH oxidase in palmitic acid-induced superoxide production and insulin secretion. Islets 5: 139–148, 2013 23817296 10.4161/isl.25459

[99] Graciano MF, Valle MM, Kowluru A, Curi R, and Carpinelli AR Regulation of insulin secretion and reactive oxygen species production by free fatty acids in pancreatic islets. Islets 3: 213–223, 2011 21750413 10.4161/isl.3.5.15935

[100] Grankvist K, Marklund SL, and Täljedal IB CuZn-superoxide dismutase, Mn-superoxide dismutase, catalase and glutathione peroxidase in pancreatic islets and other tissues in the mouse. Biochem J 199: 393–398, 1981 7041886 10.1042/bj1990393 PMC1163382

[101] Grupe M, Myers G, Penner R, and Fleig A Activation of store-operated I(CRAC) by hydrogen peroxide. Cell Calcium 48: 1–9, 2010 20646759 10.1016/j.ceca.2010.05.005 PMC2929316

[102] Guardado-Mendoza R, Davalli AM, Chavez AO, Hubbard GB, Dick EJ, Majluf-Cruz A, Tene-Perez CE, Goldschmidt L, Hart J, Perego C, Comuzzie AG, Tejero ME, Finzi G, Placidi C, La Rosa S, Capella C, Halff G, Gastaldelli A, DeFronzo RA, and Folli F Pancreatic islet amyloidosis, beta-cell apoptosis, and alpha-cell proliferation are determinants of islet remodeling in type-2 diabetic baboons. Proc Natl Acad Sci U S A 106: 13992–13997, 2009 19666551 10.1073/pnas.0906471106 PMC2729008

[103] Guo L, Inada A, Aguayo-Mazzucato C, Hollister-Lock J, Fujitani Y, Weir GC, Wright CVE, Sharma A, and Bonner-Weir S PDX1 in ducts is not required for postnatal formation of β-cells but is necessary for their subsequent maturation. Diabetes 62: 3459–3468, 2013 23775765 10.2337/db12-1833 PMC3781453

[104] Guo S, Dai C, Guo M, Taylor B, Harmon JS, Sander M, Robertson RP, Powers AC, and Stein R Inactivation of specific β cell transcription factors in type 2 diabetes. J Clin Invest 123: 3305–3316, 2013 23863625 10.1172/JCI65390 PMC3726150

[105] Gurgul-Convey E, Mehmeti I, Plötz T, Jörns A, and Lenzen S Sensitivity profile of the human EndoC-βH1 beta cell line to proinflammatory cytokines. Diabetologia 59: 2125–2133, 2016 27460666 10.1007/s00125-016-4060-y

[106] Gurzov EN and Eizirik DL Bcl-2 proteins in diabetes: mitochondrial pathways of β-cell death and dysfunction. Trends Cell Biol 21: 424–431, 2011 21481590 10.1016/j.tcb.2011.03.001

[107] Haeusler RA, McGraw TE, and Accili D Biochemical and cellular properties of insulin receptor signalling. Nat Rev Mol Cell Biol 19: 31–44, 2018 28974775 10.1038/nrm.2017.89 PMC5894887

[108] Hagman DK, Hays LB, Parazzoli SD, and Poitout V Palmitate inhibits insulin gene expression by altering PDX-1 nuclear localization and reducing MafA expression in isolated rat islets of Langerhans. J Biol Chem 280: 32413–32418, 2005 15944145 10.1074/jbc.M506000200 PMC1361267

[109] Hamaoka R, Fujii J, Miyagawa J, Takahashi M, Kishimoto M, Moriwaki M, Yamamoto K, Kajimoto Y, Yamasaki Y, Hanafusa T, Matsuzawa Y, and Taniguchi N Overexpression of the aldose reductase gene induces apoptosis in pancreatic beta-cells by causing a redox imbalance. J Biochem 126: 41–47, 1999 10393319 10.1093/oxfordjournals.jbchem.a022434

[110] Han J, Song B, Kim J, Kodali VK, Pottekat A, Wang M, Hassler J, Wang S, Pennathur S, Back SH, Katze MG, and Kaufman RJ Antioxidants complement the requirement for protein chaperone function to maintain β-cell function and glucose homeostasis. Diabetes 64: 2892–2904, 2015 25795214 10.2337/db14-1357 PMC4512214

[111] Hara T, Mahadevan J, Kanekura K, Hara M, Lu S, and Urano F Calcium efflux from the endoplasmic reticulum leads to β-cell death. Endocrinology 155: 758–768, 2014 24424032 10.1210/en.2013-1519 PMC3929724

[112] Harmon JS, Stein R, and Robertson RP Oxidative stress-mediated, post-translational loss of MafA protein as a contributing mechanism to loss of insulin gene expression in glucotoxic beta cells. J Biol Chem 280: 11107–11113, 2005 15664999 10.1074/jbc.M410345200

[113] Hauge M, Vestmar MA, Husted AS, Ekberg JP, Wright MJ, Di Salvo J, Weinglass AB, Engelstoft MS, Madsen AN, Lückmann M, Miller MW, Trujillo ME, Frimurer TM, Holst B, Howard AD, and Schwartz TW GPR40 (FFAR1)—combined Gs and Gq signaling in vitro is associated with robust incretin secretagogue action ex vivo and in vivo. Mol Metab 4: 3–14, 2015 25685685 10.1016/j.molmet.2014.10.002 PMC4314522

[114] Henquin JC Regulation of insulin secretion: a matter of phase control and amplitude modulation. Diabetologia 52: 739–751, 2009 19288076 10.1007/s00125-009-1314-y

[115] Hirata T, Kawai T, Hirose H, Tanaka K, Kurosawa H, Fujii C, Fujita H, Seto Y, Matsumoto H, and Itoh H Palmitic acid-rich diet suppresses glucose-stimulated insulin secretion (GSIS) and induces endoplasmic reticulum (ER) stress in pancreatic islets in mice. Endocr Res 41: 8–15, 2016 26167855 10.3109/07435800.2015.1038352

[116] Hoarau E, Chandra V, Rustin P, Scharfmann R, and Duvillie B Pro-oxidant/antioxidant balance controls pancreatic β-cell differentiation through the ERK1/2 pathway. Cell Death Dis 5: e1487, 2014 25341041 10.1038/cddis.2014.441 PMC4237262

[117] Hoppa MB, Collins S, Ramracheya R, Hodson L, Amisten S, Zhang Q, Johnson P, Ashcroft FM, and Rorsman P Chronic palmitate exposure inhibits insulin secretion by dissociation of Ca^2+^ channels from secretory granules. Cell Metab 10: 455–465, 2009 19945403 10.1016/j.cmet.2009.09.011 PMC2814048

[118] Hou N, Torii S, Saito N, Hosaka M, and Takeuchi T Reactive oxygen species-mediated pancreatic β-cell death is regulated by interactions between stress-activated protein kinases, p38 and c-jun N-terminal kinase, and mitogen-activated protein kinase phosphatases. Endocrinology 149: 1654–1665, 2008 18187551 10.1210/en.2007-0988

[119] Huang CF, Yang CY, Tsai JR, Wu CT, and Liu SH Lan KC Low-dose tributyltin exposure induces an oxidative stress-triggered JNK-related pancreatic β-cell apoptosis and a reversible hypoinsulinemic hyperglycemia in mice. Sci Rep 8: 5734, 2018 29636531 10.1038/s41598-018-24076-w PMC5893562

[120] Huang Y and Chang Y Regulation of pancreatic islet beta-cell mass by growth factor and hormone signaling. Prog Mol Biol Transl Sci 121: 321–349, 2014 24373242 10.1016/B978-0-12-800101-1.00010-7

[121] Huebschmann AG, Regensteiner JG, Vlassara H, and Reusch JEB Diabetes and advanced glycoxidation end products. Diabetes Care 29: 1420–1432, 2006 16732039 10.2337/dc05-2096

[122] Husted AS, Trauelsen M, Rudenko O, Hjorth SA, and Schwartz TW GPCR-mediated signaling of metabolites. Cell Metab 25: 777–796, 2017 28380372 10.1016/j.cmet.2017.03.008

[123] Igoillo-Esteve M, Marselli L, Cunha DA, Ladrière L, Ortis F, Grieco FA, Dotta F, Weir GC, Marchetti P, Eizirik DL, and Cnop M Palmitate induces a pro-inflammatory response in human pancreatic islets that mimics CCL2 expression by beta cells in type 2 diabetes. Diabetologia 53: 1395–1405, 2010 20369226 10.1007/s00125-010-1707-y

[124] Ihara Y, Toyokuni S, Uchida K, Odaka H, Tanaka T, Ikeda H, Hiai H, Seino Y, and Yamada Y Hyperglycemia causes oxidative stress in pancreatic beta-cells of GK rats, a model of type 2 diabetes. Diabetes 48: 927–932, 1999 10102716 10.2337/diabetes.48.4.927

[125] Imai Y, Dobrian AD, Morris MA, Taylor-Fishwick DA, and Nadler JL Lipids and immunoinflammatory pathways of beta cell destruction. Diabetologia 59: 673–678, 2016 26868492 10.1007/s00125-016-3890-y PMC4779407

[126] Imai Y, Fink BD, Promes JA, Kulkarni CA, Kerns RJ, and Sivitz WI Effect of a mitochondrial-targeted coenzyme Q analog on pancreatic β-cell function and energetics in high fat fed obese mice. Pharmacol Res Perspect 6: e00393, 2018 29864244 10.1002/prp2.393 PMC5980123

[127] Inoguchi T, Li P, Umeda F, Yu HY, Kakimoto M, Imamura M, Aoki T, Etoh T, Hashimoto T, Naruse M, Sano H, Utsumi H, and Nawata H High glucose level and free fatty acid stimulate reactive oxygen species production through protein kinase C-dependent activation of NAD(P)H oxidase in cultured vascular cells. Diabetes 49: 1939–1945, 2000 11078463 10.2337/diabetes.49.11.1939

[128] Inoue H, Shirakawa J, Togashi Y, Tajima K, Okuyama T, Kyohara M, Tanaka Y, Orime K, Saisho Y, Yamada T, Shibue K, Kulkarni RN, and Terauchi Y Signaling between pancreatic β cells and macrophages via S100 calcium-binding protein A8 exacerbates β-cell apoptosis and islet inflammation. J Biol Chem 293: 5934–5946, 2018 29496993 10.1074/jbc.M117.809228 PMC5912464

[129] Itoh Y, Kawamata Y, Harada M, Kobayashi M, Fujii R, Fukusumi S, Ogi K, Hosoya M, Tanaka Y, Uejima H, Tanaka H, Maruyama M, Satoh R, Okubo S, Kizawa H, Komatsu H, Matsumura F, Noguchi Y, Shinohara T, Hinuma S, Fujisawa Y, and Fujino M Free fatty acids regulate insulin secretion from pancreatic β cells through GPR40. Nature 422: 173–176, 2003 12629551 10.1038/nature01478

[130] Ivanov S, Merlin J, Lee MKS, Murphy AJ, and Guinamard RR Biology and function of adipose tissue macrophages, dendritic cells and B cells. Atherosclerosis 271: 102–110, 2018 29482037 10.1016/j.atherosclerosis.2018.01.018

[131] Ivarsson R, Quintens R, Dejonghe S, Tsukamoto K, in 't Veld P, Renström E, and Schuit FC Redox control of exocytosis: regulatory role of NADPH, thioredoxin, and glutaredoxin. Diabetes 54: 2132–2142, 2005 15983215 10.2337/diabetes.54.7.2132

[132] Iwaoka R and Kataoka K Glucose regulates Mafa transcription factor abundance and insulin gene expression by inhibiting AMP-activated protein kinase in pancreatic β-cells. J Biol Chem 293: 3524–3534, 2018 29348175 10.1074/jbc.M117.817932 PMC5846144

[133] Jabůrek M, Ježek J, Zelenka J, and Ježek P Antioxidant activity by a synergy of redox-sensitive mitochondrial phospholipase A2 and uncoupling protein-2 in lung and spleen. Int J Biochem Cell Biol 45: 816–825, 2013 23354121 10.1016/j.biocel.2013.01.010

[134] Jager J, Grémeaux T, Cormont M, Le Marchand-Brustel Y, and Tanti JF Interleukin-1β-induced insulin resistance in adipocytes through down-regulation of insulin receptor substrate-1 expression. Endocrinology 148: 241–251, 2007 17038556 10.1210/en.2006-0692 PMC1971114

[135] Janikiewicz J, Hanzelka K, Kozinski K, Kolczynska K, and Dobrzyn A Islet β-cell failure in type 2 diabetes—within the network of toxic lipids. Biochem Biophys Res Commun 460: 491–496, 2015 25843796 10.1016/j.bbrc.2015.03.153

[136] Ježek J, Dlasková A, Zelenka J, Jabůrek M, and Ježek P H_2_O_2_-activated mitochondrial phospholipase iPLA_2_γ prevents lipotoxic oxidative stress in synergy with UCP2, amplifies signaling *via* G-protein–coupled receptor GPR40, and regulates insulin secretion in pancreatic β-cells. Antioxid Redox Signal 23: 958–972, 2015 25925080 10.1089/ars.2014.6195 PMC4623989

[137] Ježek P and Hlavatá L Mitochondria in homeostasis of reactive oxygen species in cell, tissues, and organism. Int J Biochem Cell Biol 37: 2478–2503, 2005 16103002 10.1016/j.biocel.2005.05.013

[138] Ježek P, Holendová B, Garlid KD, and Jabůrek M Mitochondrial uncoupling proteins: subtle regulators of cellular redox signaling. Antioxid Redox Signal 29: 667–714, 2018 29351723 10.1089/ars.2017.7225 PMC6071544

[139] Ježek P, Jabůrek M, Holendová B, and Plecitá-Hlavatá L Fatty acid-stimulated insulin secretion vs. lipotoxicity. Molecules 23: 1483, 2018 10.3390/molecules23061483 PMC6100479 29921789

[140] This reference has been deleted

[141] Jitrapakdee S, Wutthisathapornchai A, Wallace JC, and MacDonald MJ Regulation of insulin secretion: role of mitochondrial signalling. Diabetologia 53: 1019–1032, 2010 20225132 10.1007/s00125-010-1685-0 PMC2885902

[142] Jonas JC, Sharma A, Hasenkamp W, Ilkova H, Patanè G, Laybutt R, Bonner-Weir S, and Weir GC Chronic hyperglycemia triggers loss of pancreatic β cell differentiation in an animal model of diabetes. J Biol Chem 274: 14112–14121, 1999 10318828 10.1074/jbc.274.20.14112

[143] Joseph JW, Jensen MV, Ilkayeva O, Palmieri F, Alárcon C, Rhodes CJ, and Newgard CB The mitochondrial citrate/isocitrate carrier plays a regulatory role in glucose-stimulated insulin secretion. J Biol Chem 281: 35624–35632, 2006 17001083 10.1074/jbc.M602606200

[144] Jourdan T, Godlewski G, Cinar R, Bertola A, Szanda G, Liu J, Tam J, Han T, Mukhopadhyay B, Skarulis MC, Ju C, Aouadi M, Czech MP, and Kunos G Activation of the Nlrp3 inflammasome in infiltrating macrophages by endocannabinoids mediates beta cell loss in type 2 diabetes. Nat Med 19: 1132–1140, 2013 23955712 10.1038/nm.3265 PMC4050982

[145] Jourdan T, Godlewski G, and Kunos G Endocannabinoid regulation of β-cell functions: implications for glycaemic control and diabetes. Diabetes Obes Metab 18: 549–557, 2016 26880114 10.1111/dom.12646 PMC5045244

[146] Jung IR, Choi SE, Jung JG, Lee SA, Han SJ, Kim HJ, Kim DJ, Lee KW, and Kang Y Involvement of iron depletion in palmitate-induced lipotoxicity of beta cells. Mol Cell Endocrinol 407: 74–84, 2015 25779532 10.1016/j.mce.2015.03.007

[147] Kabe Y, Ando K, Hirao S, Yoshida M, and Handa H Redox regulation of NF-κB activation: distinct redox regulation between the cytoplasm and the nucleus. Antioxid Redox Signal 7: 395–403, 2005 15706086 10.1089/ars.2005.7.395

[148] Kahancová A, Sklenář F, Ježek P, and Dlasková A Regulation of glucose-stimulated insulin secretion by ATPase inhibitory factor 1 (IF1). FEBS Lett 592: 999–1009, 2018 29380352 10.1002/1873-3468.12991

[149] Kahn SE, Hull RL, and Utzschneider KM Mechanisms linking obesity to insulin resistance and type 2 diabetes. Nature 444: 840–846, 2006 17167471 10.1038/nature05482

[150] Kamalden TA, Macgregor-Das AM, Kannan SM, Dunkerly-Eyring B, Khaliddin N, Xu Z, Fusco AP, Yazib SA, Chow RC, Duh EJ, Halushka MK, Steenbergen C, and Das S Exosomal microRNA-15a transfer from the pancreas augments diabetic complications by inducing oxidative stress. Antioxid Redox Signal 27: 913–930, 2017 28173719 10.1089/ars.2016.6844 PMC5649125

[151] Kaminski MT, Lenzen S, and Baltrusch S Real-time analysis of intracellular glucose and calcium in pancreatic beta cells by fluorescence microscopy. Biochim Biophys Acta 1823: 1697–1707, 2012 22732296 10.1016/j.bbamcr.2012.06.022

[152] Kanda H, Tateya S, Tamori Y, Kotani K, Hiasa KI, Kitazawa R, Kitazawa S, Miyachi H, Maeda S, Egashira K, and Kasuga M MCP-1 contributes to macrophage infiltration into adipose tissue, insulin resistance, and hepatic steatosis in obesity. J Clin Invest 116: 1494–1505, 2006 16691291 10.1172/JCI26498 PMC1459069

[153] Kaneto H, Kajimoto Y, Miyagawa J, Matsuoka T, Fujitani Y, Umayahara Y, Hanafusa T, Matsuzawa Y, Yamasaki Y, and Hori M Beneficial effects of antioxidants in diabetes: possible protection of pancreatic β-cells against glucose toxicity. Diabetes 48: 2398–2406, 1999 10580429 10.2337/diabetes.48.12.2398

[154] Kaneto H, Xu G, Song KH, Suzuma K, Bonner-Weir S, Sharma A, and Weir GC Activation of the hexosamine pathway leads to deterioration of pancreatic β-cell function through the induction of oxidative stress. J Biol Chem 276: 31099–31104, 2001 11390407 10.1074/jbc.M104115200

[155] Karaskov E, Scott C, Zhang L, Teodoro T, Ravazzola M, and Volchuk A Chronic palmitate but not oleate exposure induces endoplasmic reticulum stress, which may contribute to INS-1 pancreatic β-cell apoptosis. Endocrinology 147: 3398–3407, 2006 16601139 10.1210/en.2005-1494

[156] Katsuta H, Akashi T, Katsuta R, Nagaya M, Kim D, Arinobu Y, Hara M, Bonner-Weir S, Sharma AJ, Akashi K, and Weir GC Single pancreatic beta cells co-express multiple islet hormone genes in mice. Diabetologia 53: 128–138, 2010 19851748 10.1007/s00125-009-1570-x PMC2789931

[157] Kaufman BA, Li C, and Soleimanpour SA Mitochondrial regulation of β-cell function: maintaining the momentum for insulin release. Mol Aspects Med 42: 91–104, 2015 25659350 10.1016/j.mam.2015.01.004 PMC4404204

[158] Kawamori D, Shirakawa J, Liew CW, Hu J, Morioka T, Duttaroy A, Burkey B, and Kulkarni RN GLP-1 signalling compensates for impaired insulin signalling in regulating beta cell proliferation in βIRKO mice. Diabetologia 60: 1442–1453, 2017 28526921 10.1007/s00125-017-4303-6 PMC5508991

[159] Kern PA, Ranganathan S, Li C, Wood L, and Ranganathan G Adipose tissue tumor necrosis factor and interleukin-6 expression in human obesity and insulin resistance. Am J Physiol Endocrinol Metab 280: E745–E751, 2001 11287357 10.1152/ajpendo.2001.280.5.E745

[160] Kim M, Lee JS, Oh JE, Nan J, Lee H, Jung HS, Chung SS, and Park KS SIRT3 overexpression attenuates palmitate-induced pancreatic β-cell dysfunction. PLoS One 10: e0124744, 2015 25915406 10.1371/journal.pone.0124744 PMC4411148

[161] Kim MH, Kim EH, Jung HS, Yang D, Park EY, and Jun HS EX4 stabilizes and activates Nrf2 via PKCδ, contributing to the prevention of oxidative stress-induced pancreatic beta cell damage. Toxicol Appl Pharmacol 315: 60–69, 2017 27939242 10.1016/j.taap.2016.12.005

[162] Kim-Muller JY, Kim YJR, Fan J, Zhao S, Banks AS, Prentki M, and Accili D FoxO1 deacetylation decreases fatty acid oxidation in β-cells and sustains insulin secretion in diabetes. J Biol Chem 291: 10162–10172, 2016 26984405 10.1074/jbc.M115.705608 PMC4858967

[163] Kitamura YI, Kitamura T, Kruse JP, Raum JC, Stein R, Gu W, and Accili D FoxO1 protects against pancreatic β cell failure through NeuroD and MafA induction. Cell Metab 2: 153–163, 2005 16154098 10.1016/j.cmet.2005.08.004

[164] Komatsu M, Takei M, Ishii H, and Sato Y Glucose-stimulated insulin secretion: a newer perspective. J Diabetes Investig 4: 511–516, 2013 10.1111/jdi.12094 PMC4020243 24843702

[165] Koshkin V, Wang X, Scherer PE, Chan CB, and Wheeler MB Mitochondrial functional state in clonal pancreatic beta-cells exposed to free fatty acids. J Biol Chem 278: 19709–19715, 2003 12642585 10.1074/jbc.M209709200

[166] Koya D and King GL Protein kinase C activation and the development of diabetic complications. Diabetes 47: 859–866, 1998 9604860 10.2337/diabetes.47.6.859

[167] Krause MS, Mcclenaghan NH, Flatt PR, de Bittencourt PIH, Murphy C, and Newsholme P l-Arginine is essential for pancreatic β-cell functional integrity, metabolism and defense from inflammatory challenge. J Endocrinol 211: 87–97, 2011 21784771 10.1530/JOE-11-0236

[168] Kristinsson H, Bergsten P, and Sargsyan E Free fatty acid receptor 1 (FFAR1/GPR40) signaling affects insulin secretion by enhancing mitochondrial respiration during palmitate exposure. Biochim Biophys Acta 1853: 3248–3257, 2015 26408932 10.1016/j.bbamcr.2015.09.022

[169] Kugelberg E Diabetes: macrophages mediate β-cell loss in T2DM. Nat Rev Endocrinol 9: 626, 2013 10.1038/nrendo.2013.177 24019114

[170] Kulkarni RN, Brüning JC, Winnay JN, Postic C, Magnuson MA, and Kahn CR Tissue-specific knockout of the insulin receptor in pancreatic β cells creates an insulin secretory defect similar to that in type 2 diabetes. Cell 96: 329–339, 1999 10025399 10.1016/s0092-8674(00)80546-2

[171] Latour MG, Alquier T, Oseid E, Tremblay C, Jetton TL, Luo J, Lin DCH, and Poitout V GPR40 is necessary but not sufficient for fatty acid stimulation of insulin secretion in vivo. Diabetes 56: 1087–1094, 2007 17395749 10.2337/db06-1532 PMC1853382

[172] Lawson R, Maret W, and Hogstrand C Prolonged stimulation of insulin release from MIN6 cells causes zinc depletion and loss of β-cell markers. J Trace Elem Med Biol 49: 51–59, 2018 29895372 10.1016/j.jtemb.2018.04.020

[173] Laybutt DR, Hawkins YC, Lock J, Lebet J, Sharma A, Bonner-Weir S, and Weir GC Influence of diabetes on the loss of beta cell differentiation after islet transplantation in rats. Diabetologia 50: 2117–2125, 2007 17641871 10.1007/s00125-007-0749-2

[174] Lee SH, Jouihan HA, Cooksey RC, Jones D, Kim HJ, Winge DR, and McClain DA Manganese supplementation protects against diet-induced diabetes in wild type mice by enhancing insulin secretion. Endocrinology 154: 1029–1038, 2013 23372018 10.1210/en.2012-1445 PMC3578995

[175] Leguina-Ruzzi A, Průchova P, Holendová B, Ježek P, and Jabůrek M iPLA2γ ablation alters glucose homeostasis and insulin secretion in response to fatty acids. Free Radic Biol Med 112: 152–153, 2017

[176] Leloup C, Tourrel-Cuzin C, Magnan C, Karaca M, Castel J, Carneiro L, Colombani AL, Ktorza A, Casteilla L, and Pénicaud L Mitochondrial reactive oxygen species are obligatory signals for glucose-induced insulin secretion. Diabetes 58: 673–681, 2009 19073765 10.2337/db07-1056 PMC2646066

[177] Lenzen S Oxidative stress: the vulnerable β-cell. Biochem Soc Trans 36: 343–347, 2008 18481954 10.1042/BST0360343

[178] Lenzen S Chemistry and biology of reactive species with special reference to the antioxidative defence status in pancreatic β-cells. Biochim Biophys Acta Gen Subj 1861: 1929–1942, 2017 28527893 10.1016/j.bbagen.2017.05.013

[179] Lenzen S, Drinkgern J, and Tiedge M Low antioxidant enzyme gene expression in pancreatic islets compared with various other mouse tissues. Free Radic Biol Med 20: 463–466, 1996 8720919 10.1016/0891-5849(96)02051-5

[180] Li N, Karaca M, and Maechler P Upregulation of UCP2 in beta-cells confers partial protection against both oxidative stress and glucotoxicity. Redox Biol 13: 541–549, 2017 28755631 10.1016/j.redox.2017.07.012 PMC5537434

[181] Li XN, Herrington J, Petrov A, Ge L, Eiermann G, Xiong Y, Jensen MV, Hohmeier HE, Newgard CB, Garcia ML, Wagner M, Zhang BB, Thornberry NA, Howard AD, Kaczorowski GJ, and Zhou YP The role of voltage-gated potassium channels Kv2.1 and Kv2.2 in the regulation of insulin and somatostatin release from pancreatic islets. J Pharmacol Exp Ther 344: 407–416, 2013 23161216 10.1124/jpet.112.199083

[182] Lim S, Rashid MA, Jang M, Kim Y, Won H, Lee J, Woo JT, Kim YS, Murphy MP, Ali L, Ha J, and Kim SS Mitochondria-targeted antioxidants protect pancreatic β-cells against oxidative stress and improve insulin secretion in glucotoxicity and glucolipotoxicity. Cell Physiol Biochem 28: 873–886, 2011 22178940 10.1159/000335802

[183] Lin N, Chen H, Zhang H, Wan X, and Su Q Mitochondrial reactive oxygen species (ROS) inhibition ameliorates palmitate-induced INS-1 beta cell death. Endocrine 42: 107–117, 2012 22350662 10.1007/s12020-012-9633-z

[184] Liu C, Huang Y, Zhang Y, Chen X, Kong X, and Dong Y Intracellular methylglyoxal induces oxidative damage to pancreatic beta cell line INS-1 cell through Ire1α-JNK and mitochondrial apoptotic pathway. Free Radic Res 51: 337–350, 2017 28488455 10.1080/10715762.2017.1289376

[185] Liu H, Javaheri A, Godar RJ, Murphy J, Ma X, Rohatgi N, Mahadevan J, Hyrc K, Saftig P, Marshall C, McDaniel ML, Remedi MS, Razani B, Urano F, and Diwan A Intermittent fasting preserves beta-cell mass in obesity-induced diabetes via the autophagy-lysosome pathway. Autophagy 13: 1952–1968, 2017 28853981 10.1080/15548627.2017.1368596 PMC5788488

[186] Liu S, Okada T, Assmann A, Soto J, Liew CW, Bugger H, Shirihai OS, Abel ED, and Kulkarni RN Insulin signaling regulates mitochondrial function in pancreatic β-cells. PLoS One 4: e7983, 2009 19956695 10.1371/journal.pone.0007983 PMC2776992

[187] Lu H, Hao L, Li S, Lin S, Lv L, Chen Y, Cui H, Zi T, Chu X, Na L, and Sun C Elevated circulating stearic acid leads to a major lipotoxic effect on mouse pancreatic beta cells in hyperlipidaemia via a miR-34a-5p-mediated PERK/p53-dependent pathway. Diabetologia 59: 1247–1257, 2016 26969487 10.1007/s00125-016-3900-0

[188] Ly LD, Xu S, Choi SK, Ha CM, Thoudam T, Cha SK, Wiederkehr A, Wollheim CB, Lee IK, and Park KS Oxidative stress and calcium dysregulation by palmitate in type 2 diabetes. Exp Mol Med 49: e291, 2017 28154371 10.1038/emm.2016.157 PMC5336562

[189] MacDonald PE Signal integration at the level of ion channel and exocytotic function in pancreatic β-cells. Am J Physiol Endocrinol Metab 301: E1065–E1069, 2011 21934040 10.1152/ajpendo.00426.2011

[190] MacDonald PE, Salapatek AMF, and Wheeler MB Temperature and redox state dependence of native Kv2.1 currents in rat pancreatic β-cells. J Physiol 546: 647–653, 2003 12562993 10.1113/jphysiol.2002.035709 PMC2342601

[191] Maechler P Mitochondrial function and insulin secretion. Mol Cell Endocrinol 379: 12–18, 2013 23792187 10.1016/j.mce.2013.06.019

[192] Mahadevan J, Parazzoli S, Oseid E, Hertzel AV, Bernlohr DA, Vallerie SN, Liu CQ, Lopez M, Harmon JS, and Robertson RP Ebselen treatment prevents islet apoptosis, maintains intranuclear Pdx-1 and MafA levels, and preserves β-cell mass and function in ZDF rats. Diabetes 62: 3582–3588, 2013 23801580 10.2337/db13-0357 PMC3781455

[193] Mahdi T, Hänzelmann S, Salehi A, Muhammed SJ, Reinbothe TM, Tang Y, Axelsson AS, Zhou Y, Jing X, Almgren P, Krus U, Taneera J, Blom AM, Lyssenko V, Esguerra JLS, Hansson O, Eliasson L, Derry J, Zhang E, Wollheim CB, Groop L, Renström E, and Rosengren AH Secreted frizzled-related protein 4 reduces insulin secretion and is overexpressed in type 2 diabetes. Cell Metab 16: 625–633, 2012 23140642 10.1016/j.cmet.2012.10.009

[194] Mancini AD, Bertrand G, Vivot K, Carpentier É, Tremblay C, Ghislain J, Bouvier M, and Poitout V B-arrestin recruitment and biased agonism at free fatty acid receptor 1. J Biol Chem 290: 21131–21140, 2015 26157145 10.1074/jbc.M115.644450 PMC4543669

[195] Marselli L, Thorne J, Dahiya S, Sgroi DC, Sharma A, Bonner-Weir S, Marchetti P, and Weir GC Gene expression profiles of beta-cell enriched tissue obtained by laser capture microdissection from subjects with type 2 diabetes. PLoS One 5: e11499, 2010 20644627 10.1371/journal.pone.0011499 PMC2903480

[196] Martens GA, Cai Y, Hinke S, Stangé G, Van De Casteele M, and Pipeleers D Glucose suppresses superoxide generation in metabolically responsive pancreatic β cells. J Biol Chem 280: 20389–20396, 2005 15774474 10.1074/jbc.M411869200

[197] Maulucci G, Daniel B, Cohen O, Avrahami Y, and Sasson S Hormetic and regulatory effects of lipid peroxidation mediators in pancreatic beta cells. Mol Aspects Med 49: 49–77, 2016 27012748 10.1016/j.mam.2016.03.001

[198] McIntosh CHS, Widenmaier S, and Kim SJ Glucose-dependent insulinotropic polypeptide signaling in pancreatic β-cells and adipocytes. J Diabetes Investig 3: 96–106, 2012 10.1111/j.2040-1124.2012.00196.x PMC4020726 24843552

[199] Mehmeti I, Gurgul-Convey E, Lenzen S, and Lortz S Induction of the intrinsic apoptosis pathway in insulin-secreting cells is dependent on oxidative damage of mitochondria but independent of caspase-12 activation. Biochim Biophys Acta 1813: 1827–1835, 2011 21784110 10.1016/j.bbamcr.2011.06.022

[200] Mehmeti I, Lortz S, Avezov E, Jörns A, and Lenzen S ER-resident antioxidative GPx7 and GPx8 enzyme isoforms protect insulin-secreting INS-1E β-cells against lipotoxicity by improving the ER antioxidative capacity. Free Radic Biol Med 112: 121–130, 2017 28751022 10.1016/j.freeradbiomed.2017.07.021

[201] Merglen A, Theander S, Rubi B, Chaffard G, Wollheim CB, and Maechler P Glucose sensitivity and metabolism-secretion coupling studied during two-year continuous culture in INS-1E insulinoma cells. Endocrinology 145: 667–678, 2004 14592952 10.1210/en.2003-1099

[202] Mittal M, Gu XQ, Pak O, Pamenter ME, Haag D, Fuchs DB, Schermuly RT, Ghofrani HA, Brandes RP, Seeger W, Grimminger F, Haddad GG, and Weissmann N Hypoxia induces Kvchannel current inhibition by increased NADPH oxidase-derived reactive oxygen species. Free Radic Biol Med 52: 1033–1042, 2012 22222468 10.1016/j.freeradbiomed.2011.12.004

[203] Modak MA, Datar SP, Bhonde RR, and Ghaskadbi SS Differential susceptibility of chick and mouse islets to streptozotocin and its co-relation with islet antioxidant status. J Comp Physiol B 177: 247–257, 2007 17205303 10.1007/s00360-006-0126-3

[204] Modak MA, Parab PB, and Ghaskadbi SS Pancreatic islets are very poor in rectifying oxidative DNA damage. Pancreas 38: 23–29, 2009 18695629 10.1097/MPA.0b013e318181da4e

[205] Montane J, Klimek-Abercrombie A, Potter KJ, Westwell-Roper C, and Bruce Verchere C Metabolic stress, IAPP and islet amyloid. Diabetes Obes Metab 14: 68–77, 2012 22928566 10.1111/j.1463-1326.2012.01657.x

[206] Monteiro G, Horta BB, Pimenta DC, Augusto O, and Netto LES Reduction of 1-Cys peroxiredoxins by ascorbate changes the thiol-specific antioxidant paradigm, revealing another function of vitamin C. Proc Natl Acad Sci U S A 104: 4886–4891, 2007 17360337 10.1073/pnas.0700481104 PMC1829234

[207] Morgan D, Oliveira-Emilio HR, Keane D, Hirata AE, Santos da Rocha M, Bordin S, Curi R, Newsholme P, and Carpinelli AR Glucose, palmitate and pro-inflammatory cytokines modulate production and activity of a phagocyte-like NADPH oxidase in rat pancreatic islets and a clonal beta cell line. Diabetologia 50: 359–369, 2007 17151863 10.1007/s00125-006-0462-6

[208] Morita S, Shimajiri Y, Sakagashira S, Furuta M, and Sanke T Effect of exposure to non-esterified fatty acid on progressive deterioration of insulin secretion in patients with type 2 diabetes: a long-term follow-up study. Diabet Med 29: 980–985, 2012 22221293 10.1111/j.1464-5491.2011.03566.x

[209] Mukhuty A, Fouzder C, Mukherjee S, Malick C, Mukhopadhyay S, Bhattacharya S, and Kundu R Palmitate induced Fetuin-A secretion from pancreatic β-cells adversely affects its function and elicits inflammation. Biochem Biophys Res Commun 491: 1118–1124, 2017 28797566 10.1016/j.bbrc.2017.08.022

[210] Natalicchio A, Biondi G, Marrano N, Labarbuta R, Tortosa F, Spagnuolo R, D'Oria R, Carcjkjhia E, Leonardini A, Cignarelli A, Perrini S, Laviola L, and Giorgino F Long-term exposure of pancreatic β-cells to palmitate results in SREBP-1C-dependent decreases in GLP-1 receptor signaling via CREB and AKT and insulin secretory response. Endocrinology 157: 2243–2258, 2016 27035653 10.1210/en.2015-2003

[211] Newsholme P, Homem De Bittencourt PI, O' Hagan C, De Vito G, Murphy C, and Krause MS Exercise and possible molecular mechanisms of protection from vascular disease and diabetes: the central role of ROS and nitric oxide. Clin Sci (Lond) 118: 341–349, 2009 19922417 10.1042/CS20090433

[212] Newsholme P, Morgan D, Rebelato E, Oliveira-Emilio HC, Procopio J, Curi R, and Carpinelli A Insights into the critical role of NADPH oxidase(s) in the normal and dysregulated pancreatic beta cell. Diabetologia 52: 2489–2498, 2009 19809798 10.1007/s00125-009-1536-z

[213] Ng NHJ and Teo AKK Heterogeneity and cell fate fl ux in single human pancreatic islet cells. Cell Death Dis 9: 222, 2018 29445142 10.1038/s41419-018-0269-7 PMC5833390

[214] Nicholas LM, Valtat B, Medina A, Andersson L, Abels M, Mollet IG, Jain D, Eliasson L, Wierup N, Fex M, and Mulder H Mitochondrial transcription factor B2 is essential for mitochondrial and cellular function in pancreatic β-cells. Mol Metab 6: 651–663, 2017 28702322 10.1016/j.molmet.2017.05.005 PMC5485242

[215] Nicholson T, Church C, Baker DJ, and Jones SW The role of adipokines in skeletal muscle inflammation and insulin sensitivity. J Inflamm (Lond) 15: 9, 2018 29760587 10.1186/s12950-018-0185-8 PMC5944154

[216] Nishikawa T and Araki E Impact of mitochondrial ROS production in the pathogenesis of diabetes mellitus and its complications. Antioxid Redox Signal 9: 343–353, 2007 17184177 10.1089/ars.2006.1458

[217] Nti BK, Markman JL, Bertera S, Styche AJ, Lakomy RJ, Subbotin VM, Trucco M, and Zorina TD Treg cells in pancreatic lymph nodes: the possible role in diabetogenesis and β cell regeneration in a T1D model. Cell Mol Immunol 9: 455–463, 2012 23042535 10.1038/cmi.2012.36 PMC4002217

[218] Ocaña GJ, Pérez L, Guindon L, Deffit SN, Evans-Molina C, Thurmond DC, and Blum JS Inflammatory stress of pancreatic beta cells drives release of extracellular heat-shock protein 90α. Immunology 151: 198–210, 2017 28190264 10.1111/imm.12723 PMC5418464

[219] Odegaard ML, Joseph JW, Jensen MV, Lu D, Ilkayeva O, Ronnebaum SM, Becker TC, and Newgard CB The mitochondrial 2-oxoglutarate carrier is part of a metabolic pathway that mediates glucose- and glutamine-stimulated insulin secretion. J Biol Chem 285: 16530–16537, 2010 20356834 10.1074/jbc.M109.092593 PMC2878081

[220] Okada T, Liew CW, Hu J, Hinault C, Michael MD, Krtzfeldt J, Yin C, Holzenberger M, Stoffel M, and Kulkarni RN Insulin receptors in beta-cells are critical for islet compensatory growth response to insulin resistance. Proc Natl Acad Sci U S A 104: 8977–8982, 2007 17416680 10.1073/pnas.0608703104 PMC1885613

[221] Okado-Matsumoto A and Fridovich I Subcellular distribution of superoxide dismutases (SOD) in rat liver: Cu,Zn-SOD in mitochondria. J Biol Chem 276: 38388–38393, 2001 11507097 10.1074/jbc.M105395200

[222] Oliveira JM, Rebuffat SA, Gasa R, and Gomis R Targeting type 2 diabetes: lessons from a knockout model of insulin receptor substrate 2. Can J Physiol Pharmacol 92: 613–620, 2014 24977713 10.1139/cjpp-2014-0114

[223] Oslowski CM, Hara T, O'Sullivan-Murphy B, Kanekura K, Lu S, Hara M, Ishigaki S, Zhu LJ, Hayashi E, Hui ST, Greiner D, Kaufman RJ, Bortell R, and Urano F Thioredoxin-interacting protein mediates ER stress-induced β cell death through initiation of the inflammasome. Cell Metab 16: 265–273, 2012 22883234 10.1016/j.cmet.2012.07.005 PMC3418541

[224] Ota T Chemokine systems link obesity to insulin resistance. Diabetes Metab J 37: 165–172, 2013 23807918 10.4093/dmj.2013.37.3.165 PMC3689012

[225] Panse M, Kluth O, Lorza-Gil E, Kaiser G, Mühlbauer E, Schürmann A, Häring HU, Ullrich S, and Gerst F Palmitate and insulin counteract glucose-induced thioredoxin interacting protein (TXNIP) expression in insulin secreting cells via distinct mechanisms. PLoS One 13: e0198016, 2018 29813102 10.1371/journal.pone.0198016 PMC5973613

[226] Park JH, Kim SJ, Park SH, Son DG, Bae JH, Kim HK, Han J, and Song DK Glucagon-like peptide-1 enhances glucokinase activity in pancreatic β-cells through the association of Epac2 with Rim2 and Rab3A. Endocrinology 153: 574–582, 2012 22147008 10.1210/en.2011-0259

[227] Persaud SJ, Asare-Anane H, and Jones PM Insulin receptor activation inhibits insulin secretion from human islets of Langerhans. FEBS Lett 510: 225–228, 2002 11801259 10.1016/s0014-5793(01)03268-9

[228] Pi J, Bai Y, Zhang Q, Wong V, Floering LM, Daniel K, Reece JM, Deeney JT, Andersen ME, Corkey BE, and Collins S Reactive oxygen species as a signal in glucose-stimulated insulin secretion. Diabetes 56: 1783–1791, 2007 17400930 10.2337/db06-1601

[229] Plecitá-Hlavatá L and Ježek P Integration of superoxide formation and cristae morphology for mitochondrial redox signaling. Int J Biochem Cell Biol 80: 31–50, 2016 27640755 10.1016/j.biocel.2016.09.010

[230] Plötz T, Krümmel B, Laporte A, Pingitore A, Persaud SJ, Jörns A, Elsner M, Mehmeti I, and Lenzen S The monounsaturated fatty acid oleate is the major physiological toxic free fatty acid for human beta cells. Nutr Diabetes 7: 305, 2017 29269872 10.1038/s41387-017-0005-x PMC5865546

[231] Prentki M, Matschinsky FM, and Madiraju SRM Metabolic signaling in fuel-induced insulin secretion. Cell Metab 18: 162–185, 2013 23791483 10.1016/j.cmet.2013.05.018

[232] Preston AM, Gurisik E, Bartley C, Laybutt DR, and Biden TJ Reduced endoplasmic reticulum (ER)-to-Golgi protein trafficking contributes to ER stress in lipotoxic mouse beta cells by promoting protein overload. Diabetologia 52: 2369–2373, 2009 19727664 10.1007/s00125-009-1506-5

[233] Puri S, Roy N, Russ HA, Leonhardt L, French EK, Roy R, Bengtsson H, Scott DK, Stewart AF, and Hebrok M Replication confers β cell immaturity. Nat Commun 9: 485, 2018 29396395 10.1038/s41467-018-02939-0 PMC5797102

[234] Purves T, Middlemas A, Agthong S, Jude EB, Boulton AJM, Fernyhough P, and Tomlinson DR A role for mitogen-activated protein kinases in the etiology of diabetic neuropathy. FASEB J 15: 2508–2514, 2001 11689477 10.1096/fj.01-0253hyp

[235] Qian J, Gu Y, Wu C, Yu F, Chen Y, Zhu J, Yao X, Bei C, and Zhu Q Agonist-induced activation of human FFA1 receptor signals to extracellular signal-regulated kinase 1 and 2 through Gq- and Gi-coupled signaling cascades. Cell Mol Biol Lett 22: 13, 2017 28747926 10.1186/s11658-017-0043-3 PMC5522598

[236] Qiu Y, Guo M, Huang S, and Stein R Insulin gene transcription is mediated by interactions between the p300 coactivator and PDX-1, BETA2, and E47. Mol Cell Biol 22: 412–420, 2002 11756538 10.1128/MCB.22.2.412-420.2002 PMC139753

[237] Rabhi N, Salas E, Froguel P, and Annicotte JS Role of the unfolded protein response in β cell compensation and failure during diabetes. J Diabetes Res 2014: 795171, 2014 24812634 10.1155/2014/795171 PMC4000654

[238] Rardin MJ, He W, Nishida Y, Newman JC, Carrico C, Danielson SR, Guo A, Gut P, Sahu AK, Li B, Uppala R, Fitch M, Riiff T, Zhu L, Zhou J, Mulhern D, Stevens RD, Ilkayeva OR, Newgard CB, Jacobson MP, Hellerstein M, Goetzman ES, Gibson BW, and Verdin E SIRT5 regulates the mitochondrial lysine succinylome and metabolic networks. Cell Metab 18: 920–933, 2013 24315375 10.1016/j.cmet.2013.11.013 PMC4105152

[239] Rehman K and Akash MSH Mechanisms of inflammatory responses and development of insulin resistance: how are they interlinked? J Biomed Sci 23: 87, 2016 27912756 10.1186/s12929-016-0303-y PMC5135788

[240] Reinbothe TM, Ivarsson R, Li DQ, Niazi O, Jing X, Zhang E, Stenson L, Bryborn U, and Renström E Glutaredoxin-1 mediates NADPH-dependent stimulation of calcium-dependent insulin secretion. Mol Endocrinol 23: 893–900, 2009 19299446 10.1210/me.2008-0306 PMC5419284

[241] Rhodes CJ, White MF, Leahy JL, and Kahn SE Direct autocrine action of insulin on β-cells: does it make physiological sense? Diabetes 62: 2157–2163, 2013 23801714 10.2337/db13-0246 PMC3712043

[242] Rojas J, Bermudez V, Palmar J, Sofía Martínez M, Olivar LC, Nava M, Tomey D, Rojas M, Salazar J, Garicano C, and Velasco M Pancreatic beta cell death: novel potential mechanisms in diabetes therapy. J Diabetes Res 2018: 9601801, 2018 29670917 10.1155/2018/9601801 PMC5836465

[243] Roma LP, Duprez J, Takahashi HK, Gilon P, Wiederkehr A, and Jonas JC Dynamic measurements of mitochondrial hydrogen peroxide concentration and glutathione redox state in rat pancreatic β-cells using ratiometric fluorescent proteins: confounding effects of pH with HyPer but not roGFP1. Biochem J 441: 971–978, 2012 22050124 10.1042/BJ20111770

[244] Ronnebaum SM, Ilkayeva O, Burgess SC, Joseph JW, Lu D, Stevens RD, Becker TC, Sherry AD, Newgard CB, and Jensen MV A pyruvate cycling pathway involving cytosolic NADP-dependent isocitrate dehydrogenase regulates glucose-stimulated insulin secretion. J Biol Chem 281: 30593–30602, 2006 16912049 10.1074/jbc.M511908200

[245] Rorsman P and Huising MO The somatostatin-secreting pancreatic δ-cell in health and disease. Nat Rev Endocrinol 14: 404–414, 2018 29773871 10.1038/s41574-018-0020-6 PMC5997567

[246] Rutter GA, Pullen TJ, Hodson DJ, and Martinez-Sanchez A Pancreatic β-cell identity, glucose sensing and the control of insulin secretion. Biochem J 466: 203–218, 2015 25697093 10.1042/BJ20141384

[247] Saadeh M, Ferrante TC, Kane A, Shirihai O, Corkey BE, and Deeney JT Reactive oxygen species stimulate insulin secretion in rat pancreatic islets: studies using mono-oleoyl-glycerol. PLoS One 7: e30200, 2012 22272304 10.1371/journal.pone.0030200 PMC3260220

[248] Sabrautzki S, Kaiser G, Przemeck GKH, Gerst F, Lorza-Gil E, Panse M, Sartorius T, Hoene M, Marschall S, Häring HU, Hrabě de Angelis M, and Ullrich S Point mutation of Ffar1 abrogates fatty acid-dependent insulin secretion, but protects against HFD-induced glucose intolerance. Mol Metab 6: 1304–1312, 2017 29031729 10.1016/j.molmet.2017.07.007 PMC5641630

[249] Safayee S, Karbalaei N, Noorafshan A, and Nadimi E Induction of oxidative stress, suppression of glucose-induced insulin release, ATP production, glucokinase activity, and histomorphometric changes in pancreatic islets of hypothyroid rat. Eur J Pharmacol 791: 147–156, 2016 27568837 10.1016/j.ejphar.2016.08.024

[249a] Salomon RG Levuglandins and isolevuglandins: stealthy toxins of oxidative injury. Antioxid Redox Signal 7: 185–201, 2005 15650407 10.1089/ars.2005.7.185

[250] Sampson SR, Bucris E, Horovitz-Fried M, Parnas A, Kahana S, Abitbol G, Chetboun M, Rosenzweig T, Brodie C, and Frankel S Insulin increases H_2_O_2_-induced pancreatic beta cell death. Apoptosis 15: 1165–1176, 2010 20544287 10.1007/s10495-010-0517-5

[251] Sarre A, Gabrielli J, Vial G, Leverve XM, and Assimacopoulos-Jeannet F Reactive oxygen species are produced at low glucose and contribute to the activation of AMPK in insulin-secreting cells. Free Radic Biol Med 52: 142–150, 2012 22064362 10.1016/j.freeradbiomed.2011.10.437

[252] Sasson S Nutrient overload, lipid peroxidation and pancreatic beta cell function. Free Radic Biol Med 111: 102–109, 2017 27600453 10.1016/j.freeradbiomed.2016.09.003

[253] Schuit F, Flamez D, De Vos A, and Pipeleers D Glucose-regulated gene expression maintaining the glucose-responsive state of β-cells. Diabetes 51: S326–S332, 2002 12475771 10.2337/diabetes.51.2007.s326

[254] Schuit F, Moens K, Heimberg H, and Pipeleers D Cellular origin of hexokinase in pancreatic islets. J Biol Chem 274: 32803–32809, 1999 10551841 10.1074/jbc.274.46.32803

[255] Schulthess FT, Paroni F, Sauter NS, Shu L, Ribaux P, Haataja L, Strieter RM, Oberholzer J, King CC, and Maedler K CXCL10 impairs β cell function and viability in diabetes through TLR4 signaling. Cell Metab 9: 125–139, 2009 19187771 10.1016/j.cmet.2009.01.003

[256] Shida T, Kamei N, Takeda-Morishita M, Isowa K, and Takayama K Colonic delivery of docosahexaenoic acid improves impaired glucose tolerance via GLP-1 secretion and suppresses pancreatic islet hyperplasia in diabetic KK-A(y) mice. Int J Pharm 450: 63–69, 2013 23618969 10.1016/j.ijpharm.2013.04.029

[257] Shukla S and Mishra R Level of hydrogen peroxide affects expression and sub-cellular localization of Pax6. Mol Biol Rep 45: 533–544, 2018 29770908 10.1007/s11033-018-4190-z

[258] Singh S, Bhowmick DC, Pany S, Joe M, Zaghlula N, and Jeremic AM Apoptosis signal regulating kinase-1 and NADPH oxidase mediate human amylin evoked redox stress and apoptosis in pancreatic beta-cells. Biochim Biophys Acta Biomembr 1860: 1721–1733, 2018 10.1016/j.bbamem.2018.03.024 PMC6177334 29627323

[259] Sireesh D, Dhamodharan U, Ezhilarasi K, Vijay V, and Ramkumar KM Association of NF-E2 related factor 2 (Nrf2) and inflammatory cytokines in recent onset type 2 diabetes mellitus. Sci Rep 8: 5126, 2018 29572460 10.1038/s41598-018-22913-6 PMC5865120

[260] This reference has been deleted

[261] Song H, Wohltmann M, Tan M, Ladenson JH, and Turk J Group VIA phospholipase A2 mitigates palmitate-induced beta-cell mitochondrial injury and apoptosis. J Biol Chem 289: 14194–14210, 2014 24648512 10.1074/jbc.M114.561910 PMC4022886

[262] Špaček T, Pavluch V, Alán L, Capková N, Engstová H, Dlasková A, Berková Z, Saudek F, and Ježek P Nkx6.1 decline accompanies mitochondrial DNA reduction but subtle nucleoid size decrease in pancreatic islet β-cells of diabetic Goto Kakizaki rats. Sci Rep 7: 15674, 2017 29142323 10.1038/s41598-017-15958-6 PMC5688109

[263] Spacek T, Santorová J, Zacharovová K, Berková Z, Hlavatá L, Saudek F, and Jezek P Glucose-stimulated insulin secretion of insulinoma INS-1E cells is associated with elevation of both respiration and mitochondrial membrane potential. Int J Biochem Cell Biol 40: 1522–1535, 2008 18248766 10.1016/j.biocel.2007.11.015

[264] Spijker HS, Ravelli RBG, Mommaas-Kienhuis AM, Van Apeldoorn AA, Engelse MA, Zaldumbide A, Bonner-Weir S, Rabelink TJ, Hoeben RC, Clevers H, Mummery CL, Carlotti F, and De Koning EJP Conversion of mature human β-cells into glucagon-producing α-cells. Diabetes 62: 2471–2480, 2013 23569174 10.2337/db12-1001 PMC3712074

[265] Spijker HS, Song H, Ellenbroek JH, Roefs MM, Engelse MA, Bos E, Koster AJ, Rabelink TJ, Hansen BC, Clark A, Carlotti F, and De Koning EJP Loss of β-cell identity occurs in type 2 diabetes and is associated with islet amyloid deposits. Diabetes 64: 2928–2938, 2015 25918235 10.2337/db14-1752

[266] Stockwell BR, Friedmann Angeli JP, Bayir H, Bush AI, Conrad M, Dixon SJ, Fulda S, Gascón S, Hatzios SK, Kagan VE, Noel K, Jiang X, Linkermann A, Murphy ME, Overholtzer M, Oyagi A, Pagnussat GC, Park J, Ran Q, Rosenfeld CS, Salnikow K, Tang D, Torti FM, Torti SV, Toyokuni S, Woerpel KA, and Zhang DD Ferroptosis: a regulated cell death nexus linking metabolism, redox biology, and disease. Cell 171: 273–285, 2017 28985560 10.1016/j.cell.2017.09.021 PMC5685180

[267] Sun W, Liu C, Chen Q, Liu N, Yan Y, and Liu B SIRT3: a new regulator of cardiovascular diseases. Oxid Med Cell Longev 2018: 1–11, 2018 10.1155/2018/7293861 PMC5831850 29643974

[268] Supale S, Li N, Brun T, and Maechler P Mitochondrial dysfunction in pancreatic β cells. Trends Endocrinol Metab 23: 477–487, 2012 22766318 10.1016/j.tem.2012.06.002

[269] Swisa A, Avrahami D, Eden N, Zhang J, Feleke E, Dahan T, Cohen-Tayar Y, Stolovich-Rain M, Kaestner KH, Glaser B, Ashery-Padan R, and Dor Y PAX6 maintains β cell identity by repressing genes of alternative islet cell types. J Clin Invest 127: 230–243, 2017 27941241 10.1172/JCI88015 PMC5199694

[270] Swisa A, Glaser B, and Dor Y Metabolic stress and compromised identity of pancreatic beta cells. Front Genet 8: 21, 2017 28270834 10.3389/fgene.2017.00021 PMC5318414

[271] Tachibana K, Sakurai K, Yokoh H, Ishibashi T, Ishikawa K, Shirasawa T, and Yokote K Mutation in insulin receptor attenuates oxidative stress and apoptosis in pancreatic beta-cells induced by nutrition excess: reduced insulin signaling and ROS. Horm Metab Res 47: 176–183, 2015 25295420 10.1055/s-0034-1389990

[272] Takahashi HK, Santos LRB, Roma LP, Duprez J, Broca C, Wojtusciszyn A, and Jonas JC Acute nutrient regulation of the mitochondrial glutathione redox state in pancreatic β-cells. Biochem J 460: 411–423, 2014 24678915 10.1042/BJ20131361

[273] Talchai C, Xuan S, Lin HV, Sussel L, and Accili D Pancreatic β cell dedifferentiation as a mechanism of diabetic β cell failure. Cell 150: 1223–1234, 2012 22980982 10.1016/j.cell.2012.07.029 PMC3445031

[274] Taneera J, Lang S, Sharma A, Fadista J, Zhou Y, Ahlqvist E, Jonsson A, Lyssenko V, Vikman P, Hansson O, Parikh H, Korsgren O, Soni A, Krus U, Zhang E, Jing XJ, Esguerra JLS, Wollheim CB, Salehi A, Rosengren A, Renström E, and Groop L A systems genetics approach identifies genes and pathways for type 2 diabetes in human islets. Cell Metab 16: 122–134, 2012 22768844 10.1016/j.cmet.2012.06.006

[275] Tao R, Coleman MC, Pennington JD, Ozden O, Park SH, Jiang H, Kim HS, Flynn CR, Hill S, Hayes McDonald WH, Olivier AK, Spitz DR, and Gius D Sirt3-mediated deacetylation of evolutionarily conserved lysine 122 regulates MnSOD activity in response to stress. Mol Cell 40: 893–904, 2010 21172655 10.1016/j.molcel.2010.12.013 PMC3266626

[276] Tao R, Vassilopoulos A, Parisiadou L, Yan Y, and Gius D Regulation of MnSOD enzymatic activity by Sirt3 connects the mitochondrial acetylome signaling networks to aging and carcinogenesis. Antioxid Redox Signal 20: 1646–1654, 2014 23886445 10.1089/ars.2013.5482 PMC3942696

[277] Taylor BL, Liu FF, and Sander M Nkx6.1 is essential for maintaining the functional state of pancreatic beta cells. Cell Rep 4: 1262–1275, 2013 24035389 10.1016/j.celrep.2013.08.010 PMC4058003

[278] Teo AKK, Lim CS, Cheow LF, Kin T, Shapiro JA, Kang NY, Burkholder W, and Lau HH Single-cell analyses of human islet cells reveal de-differentiation signatures. Cell Death Discov 4: 14, 2018 10.1038/s41420-017-0014-5 PMC5841351 29531811

[279] Tian G, Sol ER, Xu Y, Shuai H, and Tengholm A Impaired cAMP generation contributes to defective glucose-stimulated insulin secretion after long-term exposure to palmitate. Diabetes 64: 904–915, 2015 25281428 10.2337/db14-1036

[280] Tiedge M, Lortz S, Drinkgern J, and Lenzen S Relation between antioxidant enzyme gene expression and antioxidative defense status of insulin-producing cells. Diabetes 46: 1733–1742, 1997 9356019 10.2337/diab.46.11.1733

[281] Tonooka N, Oseid E, Zhou H, Harmon JS, and Robertson RP Glutathione peroxidase protein expression and activity in human islets isolated for transplantation. Clin Transpl 21: 767–772, 2007 10.1111/j.1399-0012.2007.00736.x 17988272

[282] Tyurina YY, Shrivastava I, Tyurin VA, Mao G, Dar HH, Watkins S, Epperly M, Bahar I, Shvedova AA, Pitt B, Wenzel SE, Mallampalli RK, Sadovsky Y, Gabrilovich D, Greenberger JS, Bayır H, and Kagan VE “Only a life lived for others is worth living”: redox signaling by oxygenated phospholipids in cell fate decisions. Antioxid Redox Signal 29: 1333–1358, 2018 28835115 10.1089/ars.2017.7124 PMC6157439

[283] Uchizono Y, Alarcón C, Wicksteed BL, Marsh BJ, and Rhodes CJ The balance between proinsulin biosynthesis and insulin secretion: where can imbalance lead? Diabetes Obes Metab 9: 56–66, 2007 17919179 10.1111/j.1463-1326.2007.00774.x

[284] Uruno A, Yagishita Y, and Yamamoto M The Keap1-Nrf2 system and diabetes mellitus. Arch Biochem Biophys 566: 76–84, 2015 25528168 10.1016/j.abb.2014.12.012

[285] Velasquez C, Vasquez JS, and Balcazar N In vitro effect of fatty acids identified in the plasma of obese adolescents on the function of pancreatic β-cells. Diabetes Metab J 41: 303–315, 2017 28868828 10.4093/dmj.2017.41.4.303 PMC5583408

[286] Victor VM, Rocha M, Herance R, and Hernandez-Mijares A Oxidative stress and mitochondrial dysfunction in type 2 diabetes. Curr Pharm Des 17: 3947–3958, 2011 22188447 10.2174/138161211798764915

[287] Villard O, Brun JF, Bories L, Molinari N, Benhamou PY, Berney T, and Wojtusciszyn A The second phase of insulin secretion in nondiabetic islet-grafted recipients is altered and can predict graft outcome. J Clin Endocrinol Metab 103: 1310–1319, 2018 29319810 10.1210/jc.2017-01342

[288] Wang J, Gu W, and Chen C Knocking down insulin receptor in pancreatic beta cell lines with lentiviral-small hairpin RNA reduces glucose-stimulated insulin secretion via decreasing the gene expression of insulin, GLUT2 and Pdx1. Int J Mol Sci 19: 985, 2018 10.3390/ijms19040985 PMC5979368 29587416

[289] Wang J, Yang X, and Zhang J Bridges between mitochondrial oxidative stress, ER stress and mTOR signaling in pancreatic β cells. Cell Signal 28: 1099–1104, 2016 27185188 10.1016/j.cellsig.2016.05.007

[290] Wang X, Ge QM, Bian F, Dong Y, and Huang CM Inhibition of TLR4 protects rat islets against lipopolysaccharide-induced dysfunction. Mol Med Rep 15: 805–812, 2017 28101570 10.3892/mmr.2016.6097

[291] Wang X, Vatamaniuk MZ, Roneker CA, Pepper MP, Hu LG, Simmons RA, and Lei XG Knockouts of SOD1 and GPX1 exert different impacts on murine islet function and pancreatic integrity. Antioxid Redox Signal 14: 391–401, 2011 20586612 10.1089/ars.2010.3302 PMC3026657

[292] Wang Z, York NW, Nichols CG, and Remedi MS Pancreatic β cell dedifferentiation in diabetes and redifferentiation following insulin therapy. Cell Metab 19: 872–882, 2014 24746806 10.1016/j.cmet.2014.03.010 PMC4067979

[293] Watada H and Fujitani Y Minireview: autophagy in pancreatic β-cells and its implication in diabetes. Mol Endocrinol 29: 338–348, 2015 25633274 10.1210/me.2014-1367 PMC5414755

[294] Watkins RA, Evans-Molina C, Terrell JK, Day KH, Guindon L, Restrepo IA, Mirmira RG, Blum JS, and DiMeglio LA Proinsulin and heat shock protein 90 as biomarkers of beta-cell stress in the early period after onset of type 1 diabetes. Transl Res 168: 96.e1–106.e1, 2016 26397425 10.1016/j.trsl.2015.08.010 PMC4839287

[295] Weaver J and Taylor-Fishwick DA Relationship of NADPH oxidase-1 expression to beta cell dysfunction induced by inflammatory cytokines. Biochem Biophys Res Commun 485: 290–294, 2017 28232183 10.1016/j.bbrc.2017.02.089

[296] Weisberg SP, Hunter D, Huber R, Lemieux J, Slaymaker S, Vaddi K, Charo I, Leibel RL, and Ferrante AW CCR2 modulates inflammatory and metabolic effects of high-fat feeding. J Clin Invest 116: 115–124, 2006 16341265 10.1172/JCI24335 PMC1307559

[297] Welsh N, Margulis B, Borg LA, Wiklund HJ, Saldeen J, Flodström M, Mello MA, Andersson A, Pipeleers DG, and Hellerström C Differences in the expression of heat-shock proteins and antioxidant enzymes between human and rodent pancreatic islets: implications for the pathogenesis of insulin-dependent diabetes mellitus. Mol Med 1: 806–820, 1995 8612203 PMC2230012

[298] Westermark P, Andersson A, and Westermark GT Islet amyloid polypeptide, islet amyloid, and diabetes mellitus. Physiol Rev 91: 795–826, 2011 21742788 10.1152/physrev.00042.2009

[299] Wiederkehr A and Wollheim CB Mitochondrial signals drive insulin secretion in the pancreatic β-cell. Mol Cell Endocrinol 353: 128–137, 2012 21784130 10.1016/j.mce.2011.07.016

[300] Xiao C, Giacca A, Carpentier A, and Lewis GF Differential effects of monounsaturated, polyunsaturated and saturated fat ingestion on glucose-stimulated insulin secretion, sensitivity and clearance in overweight and obese, non-diabetic humans. Diabetologia 49: 1371–1379, 2006 16596361 10.1007/s00125-006-0211-x

[301] Xu G, Chen J, Jing G, and Shalev A Thioredoxin-interacting protein regulates insulin transcription through microRNA-204. Nat Med 19: 1141–1146, 2013 23975026 10.1038/nm.3287 PMC3835787

[302] Yang KS, Kang SW, Woo HA, Hwang SC, Chae HZ, Kim K, and Rhee SG Inactivation of human peroxiredoxin I during catalysis as the result of the oxidation of the catalytic site cysteine to cysteine-sulfinic acid. J Biol Chem 277: 38029–38036, 2002 12161445 10.1074/jbc.M206626200

[303] Yang SN, Shi Y, Yang G, Li Y, Yu J, and Berggren PO Ionic mechanisms in pancreatic β cell signaling. Cell Mol Life Sci 71: 4149–4177, 2014 25052376 10.1007/s00018-014-1680-6 PMC11113777

[304] Yao T, Fujimura T, Murayama K, Okumura K, and Seko Y Oxidative stress-responsive apoptosis inducing protein (ORAIP) plays a critical role in high glucose-induced apoptosis in rat cardiac myocytes and murine pancreatic β-cells. Cells 6: 35, 2017 10.3390/cells6040035 PMC5755494 29057797

[305] Yasui S, Mawatari K, Morizumi R, Furukawa H, Shimohata T, Harada N, Takahashi A, and Nakaya Y Hydrogen peroxide inhibits insulin-induced ATP-sensitive potassium channel activation independent of insulin signaling pathway in cultured vascular smooth muscle cells. J Med Invest 59: 36–44, 2012 22449991 10.2152/jmi.59.36

[306] Yavari A, Stocker CJ, Ghaffari S, Wargent ET, Steeples V, Czibik G, Pinter K, Bellahcene M, Woods A, Martínez De Morentin PB, Cansell C, Lam BYH, Chuster A, Petkevicius K, Nguyen-Tu MS, Martinez-Sanchez A, Pullen TJ, Oliver PL, Stockenhuber A, Nguyen C, Lazdam M, O'Dowd JF, Harikumar P, Tóth M, Beall C, Kyriakou T, Parnis J, Sarma D, Katritsis G, Wortmann DDJ, Harper AR, Brown LA, Willows R, Gandra S, Poncio V, De Oliveira Figueiredo MJ, Qi NR, Peirson SN, McCrimmon RJ, Gereben B, Tretter L, Fekete C, Redwood C, Yeo GSH, Heisler LK, Rutter GA, Smith MA, Withers DJ, Carling D, Sternick EB, Arch JRS, Cawthorne MA, Watkins H, and Ashrafian H Chronic activation of γ2 AMPK induces obesity and reduces β cell function. Cell Metab 23: 821–836, 2016 27133129 10.1016/j.cmet.2016.04.003 PMC4873618

[307] Yu H, Guo P, Xie X, Wang Y, and Chen G Ferroptosis, a new form of cell death, and its relationships with tumourous diseases. J Cell Mol Med 21: 648–657, 2017 27860262 10.1111/jcmm.13008 PMC5345622

[307a] Yu JH, Kim KH, and Kim H Role of NADPH oxidase and calcium in cerulein-induced apoptosis: involvement of apoptosis-inducing factor. Ann N Y Acad Sci 1090: 292–297, 2006 17384272 10.1196/annals.1378.031

[308] Zand H, Morshedzadeh N, and Naghashian F Signaling pathways linking inflammation to insulin resistance. Diabetes Metab Syndr 11: S307–S309, 2017 28365222 10.1016/j.dsx.2017.03.006

[309] Zhang Y, Bharathi SS, Rardin MJ, Lu J, Maringer KV, Sims-Lucas S, Prochownik EV, Gibson BW, and Goetzman ES Lysine desuccinylase SIRT5 binds to cardiolipin and regulates the electron transport chain. J Biol Chem 292: 10239–10249, 2017 28458255 10.1074/jbc.M117.785022 PMC5473227

[310] Zhang Y, Warnock GL, Ao Z, Park YJ, Safikhan N, Ghahary A, and Marzban L Amyloid formation reduces protein kinase B phosphorylation in primary islet β-cells which is improved by blocking IL-1β signaling. PLoS One 13: e0193184, 2018 29474443 10.1371/journal.pone.0193184 PMC5825069

[311] Zhao F and Wang Q The protective effect of peroxiredoxin II on oxidative stress induced apoptosis in pancreatic β-cells. Cell Biosci 2: 22, 2012 22709359 10.1186/2045-3701-2-22 PMC3461449

[312] Zheng S, Ren X, Han T, Chen Y, Qiu H, Liu W, and Hu Y Fenofibrate attenuates fatty acid-induced islet β-cell dysfunction and apoptosis via inhibiting the NF-κB/MIF dependent inflammatory pathway. Metabolism 77: 23–38, 2017 28941594 10.1016/j.metabol.2017.09.001

[313] Zhou Y, Chung ACK, Fan R, Lee HM, Xu G, Tomlinson B, Chan JCN, and Kong APS Sirt3 deficiency increased the vulnerability of pancreatic beta cells to oxidative stress-induced dysfunction. Antioxid Redox Signal 27: 962–976, 2017 28375738 10.1089/ars.2016.6859

